# Supplementary material for: Regional variation of avoidable hospitalisations in a universal health care system: a register-based cohort study from Finland 1996−2013
Source: BMJ Open. 2019 Jul 18;9(7):e029592. doi: 10.1136/bmjopen-2019-029592 (PMC6661699; doi:10.1136/bmjopen-2019-029592)
Supplement: Supplementary data [file bmjopen-2019-029592supp001.pdf]

Appendix 1. Conditions included in each category of avoidable hospitalisations

Condition	ICD-9 (Finnish version)	ICD-10	Definition notes
**Vaccine-preventable**			
Bacterial Pneumonia & Influenza	481, 4822A, 4823A, 4824A, 4829X, 483, 485, 487	J09, J10, J11, J13, J14, J15.3, J15.4, J15.7, J15.9, J16.8, J18.1, J18.8, J18.9	In any diagnosis field, do not accept if D57 is as a secondary diagnose
Immunization-Related and Preventable Conditions	032, 033, 037, 045, 055, 056, 0703A, 072, 3200A	A35, A36, A37, A80, B05, B06, B16.1, B16.9, B18.0, B18.1, B26, G00.0, M01.4	In any diagnosis field
**Chronic**			
Acute Bronchitis	4660A	J20	Only when primary diagnose, and J41-J44 or J47 as a secondary diagnose
Angina	4110, 4111, 413	I20, I24.0, I24.8, I24.9	Primary diagnose only, exclude cases with surgical procedures (A)
Asthma	493	J45, J46	Primary diagnose only
Chronic Obstructive Pulmonary Disease (COPD)	491, 492, 494, 496	J41, J42, J43, J44, J47	Primary diagnose only
Congestive Heart Failure	4029B, 428	I11.0, I50, J81	Primary diagnose only, exclude cases with cardiac procedures (B)
Diabetes Complications	2501-2508	E10.0-10.8, E11.0-E11.8, E12.0-E12.8, E13.0-E13.8, E14.0-E14.8	Primary diagnose only
Hypertension	401, 4029A	I10, I11.9	Primary diagnose only, exclude cases with cardiac procedures (B)
Iron Deficiency Anaemia	280	D50.1-D50.9	Primary diagnose only
Nutritional Deficiencies	260, 261, 262, 2680A, 2681A	E40, E41, E42, E43, E55.0, E64.3	Primary diagnose only
**Acute**			
Cellulitis	681, 682, 683, 6860A-6868C	L03, L04, L08.0, L08.8, L88, L98.0	Primary diagnose only, exclude cases with other surgical procedures than skin procedures (C)
Convulsions and Epilepsy	345, 6426, 7803A	G40, G41, O15, R56	Primary diagnose only
Dehydration and Gastroenteritis	2765A, 5583A, 5589X	E86, K52.2, K52.8, K52.9	Primary diagnose only
Dental Conditions	101, 521, 522, 523, 525, 528	A69.0, K02-K06, K08, K09.8, K09.9, K12, K13	Primary diagnose only
Gangrene	7854A+2506	R02	In any diagnosis field
Kidney and Urinary Tract Infections	5900A, 5901A	N10, N11, N12, N13.6	Primary diagnose only
Pelvic Inflammatory Disease	614	N70, N73, N74	Primary diagnose only
Perforated or Bleeding Ulcer	5310B-5310C, 5311B-5311C, 5312B-5312C, 5313B-5313C, 5314B-5314C, 5315B-5315C, 5320B-5230C, 5321B-5321C, 5322B-5322C, 5323B-5323C, 5324B-5324C, 5325B-5325C, 5340B-5340C, 5341B-5341C, 5348B-5348C	K25.0-K25.2, K25.4-K25.6, K26.0-K26.2, K26.4-K26.6, K27.0-K27.2, K27.4-K27.6, K28.0-K28.2, K28.4-K28.6	Primary diagnose only

Severe ENT infection	0340, 382, 462, 463, 465, 4721A	H66, H67, J02, J03, J06, J31.2	Primary diagnose only

A) Angina, no procedure field contains any of the following (NCSP, Nordic Classification of Surgical Procedures):

AA4WT, AA400, AA5LT, AAA-AAW, AB4AT, AB4BT, AB4CT, AB4DT, AB4FT, AB5, AB6AT, AB6BT, AB6CT, AB6DT, ABA-ABW, ACA-ACW, AD-AW, AXX90, BA3AT, BA4KT, BAA, BB2AT, BB3AT, BB4WT, BBA, BC1LT, BC2-BC4, BCA, BD-BW, CAA-CAW, CB, CCA-CCW, CD-CJ, CKA-CKW, CW, DAA-DAW, DB, DCA-DCW, DD, DEA-DEW, DFA-DFE, DFW99, DG, DHA-DLW, DMA-DPW, DQ-DW, EA, EBA-EBW, EC, EDA-EDW, EEA-EW, EF, EGA-EGW, EHA-EHW, EJ-EK, EL3AT, EL3RT, EL3YT, EL4 ,ELA-ELW, EMA-EMW, EP1AT, EP1LT, EP2AT, EP3AT, ENA-ENW, EW, FA, FBA-FBW, FCA-FCE, FD, FE1AT, FE2, FEA-FEW, FF-FL, FMA-FMW, FN1ST, FN1XT, FNA-FNW, FP-FX, GA2-GA4, GAA-GAW, GBA-GBW, GCA, GD1AT, GD1BT, GD1CT, GD1LT, GD2AT, GD2BT, GD2CT, GD3, GDA-GDW, GE1AT, GE1CT, GE1DT, GE2, GEA-GEW, GW, HA0, HA1AT, HA1DT, HA1MT, HA1ST, HA2-HA5, HAA-HAF, HW, JA1LT, JA2-JA3, JAA-JAW, JB, JCA-JCW, JDA-JDW, JE, JF3, JFA-JFW, JGA-JGW, JHA-JHW, JJ1AT, JJ2-JJ8, JJA-JJW, JK1-JK2, J3KAT, JK3BT, JK3CT, JK3FT, JK3LT, JK3NT, JK3RT, JK4-JK5, JKA-JKW, JL1-JL3, JLA-JLW, JM1AT, JM1LT, JM2, JMA-JMW, JN4LT, JW, JX1LT, JX1RT, JXA, KA2AT, KA3AT, KA3CT, KA3DT, KA3LT, KA4-KA6, KAA-KAW, KBA-KBW, KC1AT, KC2AT, KC3AT, KCA-KCW, KDA-KDW, KE1AT, KE1CT, KE2, KEA-KEW, KF1-KF7, KF8AT, KF8KT, KFA-KFW, KGA-KGW, KH1AT, KH1BT, KH1CT, KH1CT, KH1DT, KH1FT, KH1YT, KKA-KKW, KW, KX, LA1, LAA-LAW, LB1AT, LB1YT, LBA-LBW, LCA-LCW, LDA-LDW, LEA-LEW, LF-LW, LX1LT, MAA-MAW, MBA-MBW, MC-MW, NA0, NA6CT, NA7BT, NA7FT, NA7KT, NA7LT, NA9KT, NAA-NAW, NB1AT,NB1BT, NB1ZT, NB2, NBA-NBW, NCA-NCW, NDA-NDW, NEA-NEW, NFA-NFW, NGA-NGW, NHA-NHW, NJ3LT, NK1AT, NK1CT, NK1DT, NK1LT, NK2-NK3, NK4AT, NK4BT, NK5, NK6AT, NK6BT, NK6CT, NK6DT, NK6KT, NK7AT, NX, PA2ZT, PA3-PA5, PA6AT, PA8KT, PA9KT, PAA-PAW, PB1AT, PB1BT, PB1ST, PB1YT, PBA-PBW, PC2DT, PC2ET, PC5AT, PC5BT, PC5DT, PC5ET, PC5GT, PC5HT, PC5JT, PC5NT, PC5PT, PC5YT, PC6DT, PC6ET, PC6FT, PC7NT, PCA-PCW, PD1AT, PD1YT, PD2DT, PD3, PD4ST, PD5YT, PD6YT, PD7YT, PDA-PDW, PE, PG1AT, PG1BT, PG1ET-PG1LT, PG1MT-PG1VT, PG1YT-PG3YT, PG5RT-PG6NT, PGA-PGW, PH1AT, PH1FT, PH1UT, PH2ST, PH3YT, PH4AT, PH5GT, PH6GT, PH7FT, PH7UT, PH9AT, PH9ST, PH900, PHA-PHW, PJ2AT, PJ2CT, PJ2HT, PJ3-PJ4, PJ5AN, PJA-PJW, PW, PXA-PXX, QAA-QAW, QBA-QBW, QCA-QCW, QDA-QDW, QW, QXA-QXW, QX2ZT, QX3AT, QX3CT, QX3LT, QX3YT, QX4, S, TAA-TAD, TAW99, TA100, TBA-TJF, TJG10, TJJ, TJL-TJW, TK-TL, TMA-TPX, TQ, U, WXQ, WW20, WW30-WW31, WW40, WW50, WX100-WX105, WX140-WX144, WX7-WX9, XCC00, XFE00, XFN96, XFX00, XFX10, XFX20, XFX97, XJW99, XPX00, XPX04, XPX08, XPX99, XW000, XW1-XW5, XX1AT, XX1BT, XX1CT, XX1DT, XX1XT, XX2AT-XX2DT, XX2XT, XX3AT-XX3DT, XX3XT, XX4-XX7, Y, ZC-ZP, ZS-ZX, ZZ

B) Congestive heart failure and hypertension, no procedure field contains any of the following:

FEA-FEW, FFA00, FFA10-FFA30, FFA96, FFB-FFW, FG-FH, FJA00, FJA96, FJB-FJW, FKA-FKW, FK1BT, FLA00, FLA96, FLB-FLW, FMA-FMW, FN1AT, FN1BT, FN1ST, FN1XT, FN1YT, FNA-FNW, FPA-FPF, FPH-FPW, FQ, FXA00-FXN00, TFN10, TFN99, TFP00, TFP40-TFP59

C) Cellulitis, no procedure field contains any of the following:

AA4WT, AA400, AA5LT, AAA-AAW, AB4AT, AB4BT, AB4CT, AB4DT, AB4FT, AB5, AB6AT, AB6BT, AB6CT, AB6DT, ABA-ABW, ACA-ACW, AD-AW, AXX90, BA3AT, BA4KT, BAA, BB2AT, BB3AT, BB4WT, BBA, BC1LT, BC2-BC4, BCA, BD-BW, CAA-CAW, CB, CCA-CCW, CD-CJ, CKA-CKW, CW, DAA-DAW, DB, DCA-DCW, DD, DEA-DEW, DFA-DFE, DFW99, DG, DHA-DLW, DMA-DPW, DQ-DW, EA, EBA-EBW, EC, EDA-EDW, EEA-EW, EF, EGA-EGW, EHA-EHW, EJ-EK, EL3AT, EL3RT, EL3YT, EL4 ,ELA-ELW, EMA-EMW, EP1AT, EP1LT, EP2AT, EP3AT, ENA-ENW, EW, FA, FBA-FBW, FCA-FCE, FD, FE1AT, FE2, FEA-FEW, FF-FL, FMA-FMW, FN1ST, FN1XT, FNA-FNW, FP-FX, GA2-GA4, GAA-GAW, GBA-GBW, GCA, GD1AT, GD1BT, GD1CT, GD1LT, GD2AT, GD2BT, GD2CT, GD3, GDA-GDW, GE1AT, GE1CT, GE1DT, GE2, GEA-GEW, GW, HA0, HA1AT, HA1DT, HA1MT, HA1ST, HA2-HA5, HAA-HAF, HW, JA1LT, JA2-JA3, JAA-JAW, JB, JCA-JCW, JDA-JDW, JE, JF3, JFA-JFW, JGA-JGW, JHA-JHW, JJ1AT, JJ2-JJ8, JJA-JJW, JK1-JK2, J3KAT, JK3BT, JK3CT, JK3FT, JK3LT, JK3NT, JK3RT, JK4-JK5, JKA-JKW, JL1-JL3, JLA-JLW, JM1AT, JM1LT, JM2, JMA-JMW, JN4LT, JW, JX1LT, JX1RT, JXA, KA2AT, KA3AT, KA3CT, KA3DT, KA3LT, KA4-KA6, KAA-KAW, KBA-KBW, KC1AT, KC2AT, KC3AT, KCA-KCW, KDA-KDW, KE1AT, KE1CT, KE2, KEA-KEW, KF1-KF7, KF8AT, KF8KT, KFA-KFW, KGA-KGW, KH1AT, KH1BT, KH1CT, KH1CT, KH1DT, KH1FT, KH1YT, KKA-KKW, KW, KX, LA1, LAA-LAW, LB1AT, LB1YT, LBA-LBW, LCA-LCW, LDA-LDW, LEA-LEW, LF-LW, LX1LT, MAA-MAW, MBA-MBW, MC-MW, NA0, NA6CT, NA7BT, NA7FT, NA7KT, NA7LT, NA9KT, NAA-NAW, NB1AT,NB1BT, NB1ZT, NB2, NBA-NBW, NCA-NCW, NDA-NDW, NEA-NEW, NFA-NFW, NGA-NGW, NHA-NHW, NJ3LT, NK1AT, NK1CT, NK1DT, NK1LT, NK2-NK3, NK4AT, NK4BT, NK5, NK6AT, NK6BT, NK6CT, NK6DT, NK6KT, NK7AT, NX, PA2ZT, PA3-PA5, PA6AT, PA8KT, PA9KT, PAA-PAW, PB1AT, PB1BT, PB1ST, PB1YT, PBA-PBW, PC2DT, PC2ET, PC5AT, PC5BT, PC5DT, PC5ET, PC5GT, PC5HT, PC5JT, PC5NT, PC5PT, PC5YT, PC6DT, PC6ET, PC6FT, PC7NT, PCA-PCW, PD1AT, PD1YT, PD2DT, PD3, PD4ST, PD5YT, PD6YT, PD7YT, PDA-PDW, PE, PG1AT, PG1BT, PG1ET-PG1LT, PG1MT-PG1VT, PG1YT-PG3YT, PG5RT-PG6NT, PGA-PGW, PH1AT, PH1FT, PH1UT, PH2ST, PH3YT, PH4AT, PH5GT, PH6GT, PH7FT, PH7UT, PH9AT, PH9ST, PH900, PHA-PHW, PJ2AT, PJ2CT, PJ2HT, PJ3-PJ4, PJ5AN, PJA-PJW, PW, PXA-PXX, QAA25, QAB00-QAB05, QAB99, QAC, QAD20, QAE-QAF, QAG10-QAG99, QAJ, QBA25, QBB00-QBB05, QBB99, QBC,

QBD20, QBE, QBG10-QBG99, QBJ, QCA25, QCA30, QCB00-QCB05, QCB99, QCC, QCD20, QCE-QCG, QCJ, QDA25, QDB00-QDB05, QDB99, QDC, QDD20, QDE, QDG10-QDG99, QDJ, QXA25, QXB00-QXB05, QXB99, QXC, QXD20, QXE, QXG10-QXG99, QXJ, QX2ZT, QX3AT, QX3CT, QX3LT, QX3YT, QX4, S, TAA-TAD, TAW99, TA100, TBA-TJF, TJG10, TJJ, TJL-TJW, TK-TL, TMA-TPX, TQA-TQD, TQW00, TQW02, TQW30-40, TQW99, TQX00-TQX10, U, WW20, WW30-WW31, WW40, WW50, WX100-WX105, WX140-WX144, WX7-WX9, XCC00, XFE00, XFN96, XFX00, XFX10, XFX20, XFX97, XJW99, XPX00, XPX04, XPX08, XPX99, XW000, XW1-XW5, XX1AT, XX1BT, XX1CT, XX1DT, XX1XT, XX2AT-XX2DT, XX2XT, XX3AT-XX3DT, XX3XT, XX4-XX7, Y, ZC-ZP, ZS-ZX, ZZ
