# Supplementary material for: Regional variation of avoidable hospitalisations in a universal health care system: a register-based cohort study from Finland 1996−2013
Source: BMJ Open. 2019 Jul 18;9(7):e029592. doi: 10.1136/bmjopen-2019-029592 (PMC6661699; doi:10.1136/bmjopen-2019-029592)
Supplement: Author's manuscript [file bmjopen-2019-029592.draft_revisions.pdf]

BMJ Open is committed to open peer review. As part of this commitment we make the peer review history of every article we publish publicly available.

When an article is published we post the peer reviewers' comments and the authors' responses online. We also post the versions of the paper that were used during peer review. These are the versions that the peer review comments apply to.

The versions of the paper that follow are the versions that were submitted during the peer review process. They are not the versions of record or the final published versions. They should not be cited or distributed as the published version of this manuscript.

BMJ Open is an open access journal and the full, final, typeset and author-corrected version of record of the manuscript is available on our site with no access controls, subscription charges or pay-per-view fees (http://bmjopen.bmj.com).

If you have any questions on BMJ Open's open peer review process please email info.bmjopen@bmj.com

BMJ Open

Regional variation of avoidable hospitalisations in a universal health care system – a register based cohort study from Finland 1996-2013

Journal:	*BMJ Open*
Manuscript ID	bmjopen-2019-029592
Article Type:	Research
Date Submitted by the Author:	04-Feb-2019

Complete List of Authors:	Manderbacka, Kristiina; National Institute for Health and Welfare (THL), Service System Research<br>Arffman, Martti; National Institute of Health and Welfare, Health and Social Services<br>Satokangas, Markku; Terveyden ja hyvinvoinnin laitos, Service System Research; Helsingin Yliopisto, Network of Academic Health Centres and Department of General Practice and Primary Health Care<br>Keskimaki, Ilmo; National Institute of Health and Welfare, Health and Social Services; Tampereen yliopisto, Dept of Social Sciences

Keywords:	Organisation of health services < HEALTH SERVICES ADMINISTRATION & MANAGEMENT, STATISTICS & RESEARCH METHODS, PRIMARY CARE, Quality in health care < HEALTH SERVICES ADMINISTRATION & MANAGEMENT

SCHOLARONE™
Manuscripts

Regional variation of avoidable hospitalisations in a universal health care system – a register based cohort study from Finland 1996-2013

Kristiina Manderbacka[1], Martti Arffman[1], Markku Satokangas[1,2], Ilmo Keskimäki[1,3]

[1] Service System Research Unit, National Institute for Health and Welfare, P.O.Box 30, 00271 Helsinki, Finland

[2] Network of Academic Health Centres and Department of General Practice and Primary Health Care, P.O. Box 20, 00014 University of Helsinki, Finland

[3] Dept of Social Sciences, University of Tampere, 33014 Tampere, Finland

Corresponding author

Kristiina Manderbacka

Service System Research Unit, National Institute for Health and Welfare (THL), P.O.Box 30, 00271 Helsinki, Finland, tel +358 29 524 7078 email kristiina.manderbacka@thl.fi

Word count

Abstract 267

Main text 2396

For peer review only

ABSTRACT

Objectives

A persistent finding in research concerning health care and hospital use in Western countries has been regional variation in medical practices. The aim of the current study was to examine trends in avoidable hospitalisations, i.e. hospitalisations due to conditions treatable in ambulatory care in Finland in 1996-2013 and possible convergence or divergence in regional variation.

Setting

Use of hospital inpatient care in 1996–2013 among the total population in Finland.

Participants

Altogether 1 931 012 hospital inpatient care episodes among all persons residing in Finland identified from administrative registers in Finland in 1996–2013 and alive in Jan 1, 1996.

Outcome measures

We examined hospitalisations due to avoidable causes including vaccine-preventable hospitalisations, hospitalisations due to complications of chronic conditions and acute conditions treatable in ambulatory care. We calculated annual age-adjusted rates per 10,000 person years. Multilevel models were used for studying time trends in regional variation.

Results

There was a steep decline in avoidable hospitalisation rates during the study period. The decline occurred almost exclusively in hospitalisations due to chronic conditions, which diminished by about 60%. The overall correlation between hospital district intercepts and slopes in time was -0.46 ($p<0.05$) among men and -0.20 (ns) among women. Statistically highly significant diminishing

For peer review only - http://bmjopen.bmj.com/site/about/guidelines.xhtml

variation was found in hospitalisations due to chronic conditions among both men (-0.90) and women (-0.91). The variation was mainly distributed to the hospital district level.

Conclusions

The results suggest that chronic conditions are managed better in primary care in the whole country than before. Further research is needed on whether this is the case or whether this has more to do with supply of hospital care.

For peer review only

Strengths and limitations of this study

- Our register based data covers the total population in Finland identified from register sources in Finland over a 18-year follow-up period.
- We used two indicators of region of residence that are directly connected to the organisation of public health care for the residents enabling us to disentangle the association of each organisational level to regional variations in hospitalisation rates.
- Multilevel models enabled us to capture the hierarchical nature of the data (health centres nested in hospital districts) allowing us to analyse the association more efficiently.
- We were not able to control for need for care i.e. disease prevalence or severity, and thus cannot estimate what part of regional differences in avoidable hospitalisations would be warranted due to differences in disease prevalence and/or severity.

For peer review only - http://bmjopen.bmj.com/site/about/guidelines.xhtml

BACKGROUND OF THE STUDY

A persistent finding in research concerning health care and hospital use in Western countries has been regional variation in medical practices. While there are regional variations within countries in disease prevalence, earlier studies have suggested that at least some of the variation in medical care is unwarranted, i.e. within country variation does not disappear when need is accounted for [1]. There is also a need to examine whether medical practice variation changes in time [1]. In a relatively recent systematic review Corallo et al [2] report a large number of studies examining medical practice variation from OECD countries covering regional differences in ambulatory care, medical admissions, elective surgery, as well as emergency hospitalisations and avoidable hospitalisations, i.e. hospitalisations due to conditions treatable in ambulatory care. Avoidable hospitalisations are commonly used as an indirect indicator of access to and quality of health care [3,4]. Earlier studies use slightly different lists of conditions, but generally three types of conditions are examined: admissions that should be avoided with timely access to preventive measures, ambulatory treatment of acute conditions and managing of certain chronic disease in an ambulatory setting.

While there are earlier studies examining regional differences in avoidable hospitalisations, many examine only some parts of the country in question [5-10], specific age groups [5,7,10], are cross-sectional [5,9,11] and/or study these hospitalisations in specific patient groups, e.g. persons with diabetes [12]. Further, some countries publish regularly Atlases of regional differences in medical practices, some of which also report avoidable hospitalisations; see e.g. [13,14].

The aim of this register-based study was to examine trends in avoidable hospitalisations in 1996-2013, possible convergence or divergence in regional variation and on what level of health care the variation occurs. The Finnish health-care system provides a good case for examining avoidable

For peer review only - http://bmjopen.bmj.com/site/about/guidelines.xhtml

hospitalisations as the system is universal in coverage and therefore, in general, supports equal access to both primary and specialized health-care according to need [15]. The system is mainly financed by tax revenues and user-fees are generally low. The system also supports evidence based care as there are accepted guidelines for the treatment of altogether 106 conditions in the National Current Care guidelines system [16]. Ambulatory services are primarily provided by the public sector and financed through taxation, but there have been difficulties in access in some areas. There are 21 hospital districts in Finland providing specialist services to the residents owned, funded and managed by municipalities.

MATERIALS AND METHODS

The data

We obtained avoidable hospitalisations for the total Finnish population aged 20 years or older in 1996-2013 from the Finnish hospital discharge register, maintained by the National Institute for Health and Welfare (THL). The linkages were performed by competent authorities and the research group received anonymized data. Ethical approval for the study was received from the Research Ethics Committee of THL (decision #7/2013 §586).

We used the UK definition of avoidable hospitalisations [3,4] with an inclusion of ICD10 codes J18.9 and J09 (pneumonia). Avoidable hospitalisations were classified into three subcategories: acute, chronic, and vaccine-preventable (for diagnoses included, their ICD10-codes, see appendix 1). Hospitalisations were combined if they occurred within one day from each other. Data on hospitalisations were linked into demographic data obtained from Statistics Finland.

We used two indicators of region of residence: (1) health centre area (single municipality or co-operation area of municipalities) that is responsible for organising primary health care for the

For peer review only - http://bmjopen.bmj.com/site/about/guidelines.xhtml

For peer review only

residents and (2) hospital district (owned by the federations of municipalities), which is responsible for organising the public specialist services in the area. Publicly funded hospitals produce ca 95% of all specialised inpatient services in Finland. The municipality of residence was linked for each individual at the end of the year preceding hospitalisation. The small autonomous region of Åland was excluded from the analyses. Age at hospitalization was classified into 5-year age brackets with those 85 years or older as the oldest group. Those living permanently in institutions were excluded from analyses. Total Finnish population was used as the population at risk in the analyses. All analyses were conducted separately for men and women.

Statistical methods

We calculated national age-standardised rates for each category of avoidable hospitalisation per 10 000 person years with direct standardisation method with the European standard population as standard population. We examined possible convergence of hospitalization rates at the hospital district level. First, we calculated autocorrelations in each of the categories and hospital districts to study consistency in hospitalisation rates and their suitability for further analysis. Next, we performed two-level models to receive annual z-scores of the age-standardised hospitalization rates in each hospital district and category adjusted for polynomial function of time with normally distributed random intercept and slope terms at the hospital district level. Z-scores were then used to examine the hospitalization rates from different categories of avoidable hospitalisations in a comparable scale. Multilevel models were applied to manage the hierarchical nature of the data and polynomial function of time to capture the general national trend in each category. We calculated correlation between the random intercept and slope terms in these models to obtain an estimate of convergence in hospitalization rates. A statistically significant negative correlation was interpreted as a decrease of regional variation in time with higher than average random

For peer review only - http://bmjopen.bmj.com/site/about/guidelines.xhtml

intercepts linked with a larger than average decrease in random slopes and vice versa. A similar procedure was fitted to all hospitalization rates due to avoidable causes simultaneously where polynomial function of time was adjusted for each category of avoidable hospitalisations to calculate an estimate for possible general convergence of hospitalisation rates [1,17]. In a separate analysis, we used three-level Poisson regression models to examine whether variation between avoidable hospitalisations was mainly in the hospital district or health centre level. In these models, year was controlled as a continuous variable and age group as a categorical variable with hospital districts and health centres as random variables. In order to assess possible changes in the levels of regional variation, we estimated models separately for the years 1996−1999 and 2010−2013. Wald test was used to assess the statistical significance of random components in the models. Variation in age-adjusted avoidable hospitalization rates in health centre areas was illustrated with scatter plots in the last four years of study period.

Patient and public involvement

As the study was based on anonymized data from administrative registers, patients were not involved in the study. The results will be disseminated to the public through summary articles after the original studies have been published.

RESULTS

There were altogether 983 966 avoidable hospital admissions among men and 947 046 among women during the study period. More than half of the hospitalisations were due to chronic conditions.

[Figure 1 around here]

For peer review only - http://bmjopen.bmj.com/site/about/guidelines.xhtml

Figure 1 presents the national rates of avoidable hospitalisations. Avoidable hospitalisations decreased consistently during the study period. The total rate of avoidable hospitalisations was 535 per 10 000 person years (py) among men in 1996 and 306 per 10 000 py in 2013. Among women the rates were 340 in 1996 and 221 in 2013. The rates were larger among men throughout the study period in each of the avoidable hospitalisation categories. While the rates remained relatively stable in hospitalisations due to vaccine-preventable conditions and acute conditions, there was a large decrease in hospitalisations for chronic conditions both among men and among women during the study period.

In the analyses of regional trends, we first inspected autocorrelations for the consistency of hospitalisation rates in each category of avoidable hospitalisations both in the health centre and in the hospital district level. We found autocorrelations in health centre level vary a lot suggesting that health centre level was not suitable for the analysis of convergence. The hospital district level autocorrelations were relatively low among men in vaccine-preventable hospitalisations, averaging 0.22, among women the autocorrelations were higher, averaging 0.35. In hospitalisations due to acute conditions, the results were rather similar with autocorrelations averaging 0.44 among men and 0.36 among women. The autocorrelations for hospitalisations due to chronic conditions were higher, with average of 0.78 among both men and women. These numbers suggest that hospital district patterns in hospitalisations were relatively consistent in time.

In multilevel analysis (Table 1), the correlation between hospital district intercepts and slopes of time was negative among men suggesting decreasing variation when all categories of avoidable hospitalisations were examined together.

For peer review only - http://bmjopen.bmj.com/site/about/guidelines.xhtml

Table 1. Multilevel model based correlation between hospital district intercepts and slopes in time between 1996 and 2013

	Men	Women
Preventable	-0.02 ns	0.44 ns
Chronic conditions	-0.90***	-0.91***
Acute conditions	-0.45*	0.01 ns

* p<0.05, **p<0.01, *** p<0.001, ns statistically non-significant

Among women, no such correlation was found. The correlations were -.0.46 (p<0.05) among men and -0.22 (ns) among women. However, when examining categories of avoidable hospitalisation separately, negative correlations indicating convergence of hospitalisation rates in hospital districts were found in rates due to chronic conditions among both genders and in rates due to acute conditions among men. In other categories, no correlations indicating convergence (or divergence) were found. The results were mostly similar when health centre level was used in the analysis (data not shown).

Finally we examined to which regional level (health centre level vs. hospital district level) the variation was mainly distributed in each of the categories of avoidable hospitalisations in the beginning (1996−1999) and in the end (2010−2013) of the study period. We examined these two periods due to decrease in avoidable hospitalisation rates during the study period (Table 2).

For peer review only - http://bmjopen.bmj.com/site/about/guidelines.xhtml

Table 2. The distribution of variance in avoidable hospitalisations to health centre (HC) and hospital district (HD) level in Finland in 1996-1999 and 2010-2013 (Poisson multilevel models controlling for year as a continuous variable and age as a categorical variable; estimates of variance components and their standard errors)

			1996-1999		2010-2013	
	Category	Level	Estimate	s.e.	Estimate	s.e.
Men	Preventable	HD	0.024**	0.010	0.027**	0.010
		HC	0.022***	0.004	0.024***	0.004
	Chronic	HD	0.051**	0.018	0.064**	0.023
		HC	0.019***	0.003	0.027***	0.004
	Acute	HD	0.025**	0.009	0.039**	0.015
		HC	0.015***	0.003	0.024***	0.004
Women	Preventable	HD	0.032**	0.013	0.033**	0.013
		HC	0.033***	0.006	0.029***	0.005
	Chronic	HD	0.054**	0.020	0.059**	0.021
		HC	0.031***	0.005	0.033***	0.005
	Acute	HD	0.026**	0.010	0.043**	0.016
		HC	0.019***	0.003	0.029***	0.005

*p<0.05, ** p<0.01, *** p<0.001

In hospitalisations due to vaccine-preventable conditions the variance was equally distributed to both levels in the late 1990s and remained similar at the end of the study period among both men and women. In hospitalisations due to chronic conditions the variance was distributed mainly to the hospital district level. Similar, but smaller variation was found in hospitalisations due to acute conditions. However, health centre level variance was also statistically significant in all categories of avoidable hospitalisations among both men and women.

[Figure 2 around here]

For peer review only - http://bmjopen.bmj.com/site/about/guidelines.xhtml

Figure 2 illustrates health centre level variation in age-adjusted rates in the three categories of avoidable hospitalisations in the end of the study period (2010-2013). In the figure, each dot represents one health centre. The variation was, in general, smaller among women compared to men in each of the categories. In vaccine-preventable hospitalisations the health centre rates were close to each other with some outliers especially towards the higher end. In hospitalisations due to acute conditions, the variation was similar but smaller. In hospitalisations due to chronic conditions the variation was large, and especially among men.

DISCUSSION

Overview of results

We found a decreasing trend in avoidable hospitalisations from the mid-1990s to the early 2010s. This trend was clear in avoidable hospitalisations due to chronic conditions, but not in vaccine-preventable hospitalisations or hospitalisations due to acute conditions. There were large hospital district variations in avoidable hospitalisations due to chronic conditions. Also, in multilevel analyses the correlation between hospital district intercepts and slopes was negative in these hospitalisations, indicating decreasing hospital district variation and convergence between hospital districts. Our result concerning relatively large regional variation in avoidable hospitalisations is in line with earlier results from other countries [5-9,11,18,19] and in different categories of avoidable hospitalisations [13,14]. Several reasons have been presented to regional variation, including differences in health status, rurality, and socioeconomic composition between regions [6-9,20,21], as well as access to and accessibility of primary health care and supply of hospital beds [9,10,18,19].

Earlier studies have mainly been cross-sectional and have not examined trends in avoidable hospitalisations, but our result concerning decreasing variation is in line with results from Canada

For peer review only - http://bmjopen.bmj.com/site/about/guidelines.xhtml

[11] as well as earlier Finnish results on hospitalisations due to complications among persons with diabetes [12]. Further research is needed to study the effects of differences in the regional sociodemographic composition of the population and supply side factors to avoidable hospitalisations. On the supply side, an important question is whether the variation is driven by access to and accessibility of primary care or supply of hospital inpatient care. Our results on larger variation in hospital district level in hospitalisations due to chronic conditions support previous results [6,22], which suggest that different categories of avoidable hospitalisations should be analysed separately.

Methodological considerations

A major strength of our study was that we were able to use individual level data of the total population of Finland over a period of 18 years. The data concerning avoidable hospitalisations were derived from the Finnish Hospital Discharge Register, which has been found to have, in general, good quality and coverage [23]. Use of multilevel models enabled us to capture the hierarchical nature of the data (health centres nested in hospital districts) and to analyse the association more efficiently.

Similarly to many earlier studies we were unable to control for need for care i.e. disease prevalence or severity, and thus cannot directly evaluate what part of regional differences in avoidable hospitalisations would be warranted due to differences in disease prevalence and/or severity. It is still unclear whether the decrease in rates of chronic category and their regional variation occurred mainly due to the development of primary care or whether it is due to overall reduction in hospital beds. This requires further studies assessing both the development of hospital supply and number of total hospitalisations.

Conclusions

For peer review only - http://bmjopen.bmj.com/site/about/guidelines.xhtml

For peer review only

Large but decreasing regional variation was found in our study in avoidable hospitalisations due to chronic conditions. These hospitalisations occur mainly due to complications of the underlying disease. The result may suggest that chronic conditions are better managed in primary care in the whole country than before. Further research is needed on whether this is the case or whether this has more to do with supply of hospital care.

For peer review only

For peer review only - http://bmjopen.bmj.com/site/about/guidelines.xhtml

Funding

The study was funded by the Strategic Research Council (grants 312 703 and 312 708) and the Medical Research Council (grant 277 939) at the Academy of Finland, but the Academy was not involved in study design, data collection, findings or decision to publish.

Authors' contributions

KM contributed to the conception and design of the study, planning of analyses, and drafted the manuscript. MA contributed to the conception and design of the study, performed the statistical analyses and took part in the revision of the manuscript for important intellectual content. MS contributed to the conception and design of the study, planning of analyses, and took part in the revision of the manuscript for important intellectual content. IK contributed to the conception and design of the study, planning of analyses and took part in the revision of the manuscript for important intellectual content. All authors have read and approved the final manuscript.

Competing interests

The authors declare no conflicts of interest that could have biased the work.

Data sharing statement

Due to Finnish data protection legislation, no further data is available.

For peer review only

For peer review only - http://bmjopen.bmj.com/site/about/guidelines.xhtml

Bibliography

1 Westert GP, Groenewegen PP, Boshuizen HC, et al. Medical practice variations in hospital care; time trends of a spatial phenomenon. *Health Place* 2004;**10**:215-220.

2 Corallo AN, Croxford R, Goodman DC, et al. A systematic review of medical practice variation in OECD countries. *Health Policy* 2014;**114**:5-14.

3 Purdy S, Griffin T, Salisbury C, et al. Ambulatory care sensitive conditions: terminology and disease coding need to be more specific to aid policy makers and clinicians. *Public Health* 2009;**123**:169-173.

4 Tian Y, Dixon A, Gao H. Data briefing: Emergency hospital admissions for ambulatory care-sensitive conditions: identifying the potential for reductions. London: *The Kings Fund* 2012.

5 Magan P, Otero A, Alberquilla A, et al. Geographic variations in avoidable hospitalizations in the elderly, in a health system with universal coverage. *BMC Health Serv Res* 2008;**8**:42.

6 Falster MO, Jorm LR, Douglas KA, et al. Sociodemographic and health characteristics, rather than primary care supply, are major drivers of geographic variation in preventable hospitalizations in Australia. *Med Care* 2015;**53**:436-445.

7 Hale N, Probst J, Robertson A. Rural Area Deprivation and Hospitalizations Among Children for Ambulatory Care Sensitive Conditions. *J Community Health* 2016;**41**:451-460.

8 Gusmano MK, Weisz D, Rodwin VG, et al. Disparities in access to health care in three French regions. *Health Policy* 2014;**114**:31-40.

For peer review only

For peer review only - http://bmjopen.bmj.com/site/about/guidelines.xhtml

9 Probst JC, Laditka JN, Laditka SB. Association between community health center and rural health clinic presence and county-level hospitalization rates for ambulatory care sensitive conditions: an analysis across eight US states. *BMC Health Serv Res* 2009;**9**:134.

10 Butler DC, Thurecht L, Brown L, et al. Social exclusion, deprivation and child health: a spatial analysis of ambulatory care sensitive conditions in children aged 0-4 years in Victoria, Australia. *Soc Sci Med* 2013;**94**:9-16.

11 Sanchez M, Vellanky S, Herring J, et al. Variations in Canadian rates of hospitalization for ambulatory care sensitive conditions. *Healthc Q* 2008;**11**:20-22.

12 Manderbacka K, Arffman M, Lumme S, et al. Regional trends in avoidable hospitalisations due to complications among population with diabetes in Finland in 1996-2011: a register-based cohort study. *BMJ Open* 2016;**6**:011620.

13 Australian Commission of Quality and Safety in Health Care. The Second Australian Atlas of Healthcare Variation. Sydney: *Australian Commission on Quality and Safety in Health Care* 2017.

14 NHS Rightcare. NHS Atlas of Variation in Healthcare 2015. Leeds: *NHS Rightcare* 2015.

15 Vuorenkoski L, Mladovsky P, Mossialos E. Finland: Health system review. *Health Systems in Transition* 2008;**10(4)**:168.

16 The Finnish Medical Society Duodecim. Current Care Guidelines. 2018. https://www.duodecim.fi/english/products/current-care-guidelines/ (accessed 11.1.2018).

17 Snijders T. Analysis of longitudinal data using the hierarchical linear model. *Quality and quantity* 1995;**30**:405-426.

For peer review only - http://bmjopen.bmj.com/site/about/guidelines.xhtml

18 Sexton E, Bedford D. GP supply, deprivation and emergency admission to hospital for COPD and diabetes complications in counties across Ireland: an exploratory analysis. *Ir J Med Sci* 2016;**185**:453-461.

19 Weeks WB, Ventelou B, Paraponaris A. Rates of admission for ambulatory care sensitive conditions in France in 2009-2010: trends, geographic variation, costs, and an international comparison. *Eur J Health Econ* 2016;**17**:453–470.

20 Löfqvist T, Burström B, Walander A, et al. Inequalities in avoidable hospitalisation by area income and the role of individual characteristics: a population-based register study in Stockholm County, Sweden. 2013 Sep 30. *BMJ Qual Saf* 2013;**Sep 30**:001715. [Epub ahead of print].

21 Ansari Z, Haider SI, Ansari H, et al. Patient characteristics associated with hospitalisations for ambulatory care sensitive conditions in Victoria, Australia. *BMC Health Serv Res* 2012;**12**:475.

22 Saver BG, Wang CY, Dobie SA, et al. The central role of comorbidity in predicting ambulatory care sensitive hospitalizations. *Eur J Public Health* 2014;**24**:66-72.

23 Sund R. Quality of the Finnish Hospital Discharge Register: a systematic review. *Scan J Public Health* 2012;**40**:505-515.

For peer review only

For peer review only - http://bmjopen.bmj.com/site/about/guidelines.xhtml

Figure legends

Figure 1. Trends in avoidable hospitalisations among men and women by category in Finland 1996-2013, age-standardised rates per 10 000 person years

Figure 2. Health centre level variation in avoidable hospitalisations among men and women in 2010-2013, age-adjusted rates per 10 000 person years

For peer review only

For peer review only - http://bmjopen.bmj.com/site/about/guidelines.xhtml

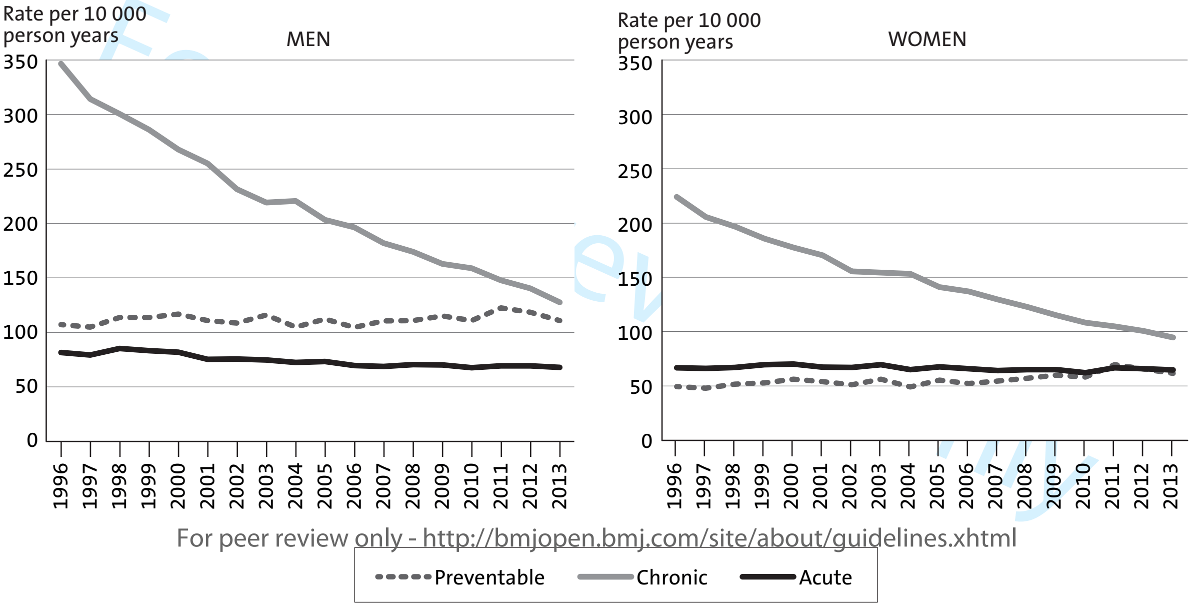

For peer review only - http://bmjopen.bmj.com/site/about/guidelines.xhtml

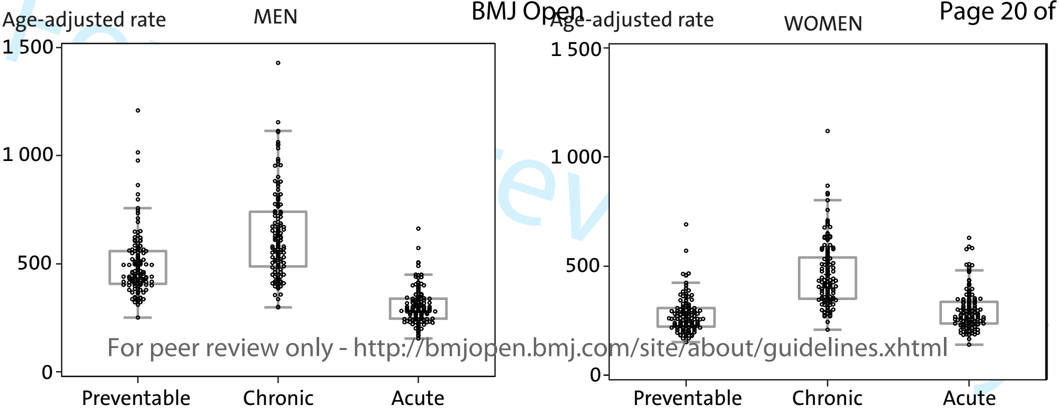

For peer review only - http://bmjopen.bmj.com/site/about/guidelines.xhtml

Appendix 1. Conditions included in each category of avoidable hospitalisations

Condition	ICD-9 (Finnish version)	ICD-10	Definition notes
**Vaccine-preventable**			
Bacterial Pneumonia & Influenza	481, 4822A, 4823A, 4824A, 4829X, 483, 485, 487	J09, J10, J11, J13, J14, J15.3, J15.4, J15.7, J15.9, J16.8, J18.1, J18.8, J18.9	In any diagnosis field, do not accept if D57 is as a secondary diagnose
Immunization-Related and Preventable Conditions	032, 033, 037, 045, 055, 056, 0703A, 072, 3200A	A35, A36, A37, A80, B05, B06, B16.1, B16.9, B18.0, B18.1, B26, G00.0, M01.4	In any diagnosis field
**Chronic**			
Acute Bronchitis	4660A	J20	Only when primary diagnose, and J41-J44 or J47 as a secondary diagnose
Angina	4110, 4111, 413	I20, I24.0, I24.8, I24.9	Primary diagnose only, exclude cases with surgical procedures (A)
Asthma	493	J45, J46	Primary diagnose only
Chronic Obstructive Pulmonary Disease (COPD)	491, 492, 494, 496	J41, J42, J43, J44, J47	Primary diagnose only
Congestive Heart Failure	4029B, 428	I11.0, I50, J81	Primary diagnose only, exclude cases with cardiac procedures (B)
Diabetes Complications	2501-2508	E10.0-10.8, E11.0-E11.8, E12.0-E12.8, E13.0-E13.8, E14.0-E14.8	Primary diagnose only
Hypertension	401, 4029A	I10, I11.9	Primary diagnose only, exclude cases with cardiac procedures (B)
Iron Deficiency Anaemia	280	D50.1-D50.9	Primary diagnose only
Nutritional Deficiencies	260, 261, 262, 2680A, 2681A	E40, E41, E42, E43, E55.0, E64.3	Primary diagnose only
**Acute**			
Cellulitis	681, 682, 683, 6860A-6868C	L03, L04, L08.0, L08.8, L88, L98.0	Primary diagnose only, exclude cases with other surgical procedures than skin procedures (C)
Convulsions and Epilepsy	345, 6426, 7803A	G40, G41, O15, R56	Primary diagnose only
Dehydration and Gastroenteritis	2765A, 5583A, 5589X	E86, K52.2, K52.8, K52.9	Primary diagnose only
Dental Conditions	101, 521, 522, 523, 525, 528	A69.0, K02-K06, K08, K09.8, K09.9, K12, K13	Primary diagnose only
Gangrene	7854A+2506	R02	In any diagnosis field
Kidney and Urinary Tract Infections	5900A, 5901A	N10, N11, N12, N13.6	Primary diagnose only
Pelvic Inflammatory Disease	614	N70, N73, N74	Primary diagnose only
Perforated or Bleeding Ulcer	5310B-5310C, 5311B-5311C, 5312B-5312C, 5313B-5313C, 5314B-5314C, 5315B-5315C, 5320B-5230C, 5321B-5321C, 5322B-5322C, 5323B-5323C, 5324B-5324C, 5325B-5325C, 5340B-5340C, 5341B-5341C, 5348B-5348C	K25.0-K25.2, K25.4-K25.6, K26.0-K26.2, K26.4-K26.6, K27.0-K27.2, K27.4-K27.6, K28.0-K28.2, K28.4-K28.6	Primary diagnose only

For peer review only

Severe ENT infection	0340, 382, 462, 463, 465, 4721A	H66, H67, J02, J03, J06, J31.2	Primary diagnose only

For peer review only

A) Angina, no procedure field contains any of the following (NCSP, Nordic Classification of Surgical Procedures):

AA4WT, AA400, AA5LT, AAA-AAW, AB4AT, AB4BT, AB4CT, AB4DT, AB4FT, AB5, AB6AT, AB6BT, AB6CT, AB6DT, ABA-ABW, ACA-ACW, AD-AW, AXX90, BA3AT, BA4KT, BAA, BB2AT, BB3AT, BB4WT, BBA, BC1LT, BC2-BC4, BCA, BD-BW, CAA-CAW, CB, CCA-CCW, CD-CJ, CKA-CKW, CW, DAA-DAW, DB, DCA-DCW, DD, DEA-DEW, DFA-DFE, DFW99, DG, DHA-DLW, DMA-DPW, DQ-DW, EA, EBA-EBW, EC, EDA-EDW, EEA-EW, EF, EGA-EGW, EHA-EHW, EJ-EK, EL3AT, EL3RT, EL3YT, EL4 ,ELA-ELW, EMA-EMW, EP1AT, EP1LT, EP2AT, EP3AT, ENA-ENW, EW, FA, FBA-FBW, FCA-FCE, FD, FE1AT, FE2, FEA-FEW, FF-FL, FMA-FMW, FN1ST, FN1XT, FNA-FNW, FP-FX, GA2-GA4, GAA-GAW, GBA-GBW, GCA, GD1AT, GD1BT, GD1CT, GD1LT, GD2AT, GD2BT, GD2CT, GD3, GDA-GDW, GE1AT, GE1CT, GE1DT, GE2, GEA-GEW, GW, HA0, HA1AT, HA1DT, HA1MT, HA1ST, HA2-HA5, HAA-HAF, HW, JA1LT, JA2-JA3, JAA-JAW, JB, JCA-JCW, JDA-JDW, JE, JF3, JFA-JFW, JGA-JGW, JHA-JHW, JJ1AT, JJ2-JJ8, JJA-JJW, JK1-JK2, J3KAT, JK3BT, JK3CT, JK3FT, JK3LT, JK3NT, JK3RT, JK4-JK5, JKA-JKW, JL1-JL3, JLA-JLW, JM1AT, JM1LT, JM2, JMA-JMW, JN4LT, JW, JX1LT, JX1RT, JXA, KA2AT, KA3AT, KA3CT, KA3DT, KA3LT, KA4-KA6, KAA-KAW, KBA-KBW, KC1AT, KC2AT, KC3AT, KCA-KCW, KDA-KDW, KE1AT, KE1CT, KE2, KEA-KEW, KF1-KF7, KF8AT, KF8KT, KFA-KFW, KGA-KGW, KH1AT, KH1BT, KH1CT, KH1CT, KH1DT, KH1FT, KH1YT, KKA-KKW, KW, KX, LA1, LAA-LAW, LB1AT, LB1YT, LBA-LBW, LCA-LCW, LDA-LDW, LEA-LEW, LF-LW, LX1LT, MAA-MAW, MBA-MBW, MC-MW, NA0, NA6CT, NA7BT, NA7FT, NA7KT, NA7LT, NA9KT, NAA-NAW, NB1AT,NB1BT, NB1ZT, NB2, NBA-NBW, NCA-NCW, NDA-NDW, NEA-NEW, NFA-NFW, NGA-NGW, NHA-NHW, NJ3LT, NK1AT, NK1CT, NK1DT, NK1LT, NK2-NK3, NK4AT, NK4BT, NK5, NK6AT, NK6BT, NK6CT, NK6DT, NK6KT, NK7AT, NX, PA2ZT, PA3-PA5, PA6AT, PA8KT, PA9KT, PAA-PAW, PB1AT, PB1BT, PB1ST, PB1YT, PBA-PBW, PC2DT, PC2ET, PC5AT, PC5BT, PC5DT, PC5ET, PC5GT, PC5HT, PC5JT, PC5NT, PC5PT, PC5YT, PC6DT, PC6ET, PC6FT, PC7NT, PCA-PCW, PD1AT, PD1YT, PD2DT, PD3, PD4ST, PD5YT, PD6YT, PD7YT, PDA-PDW, PE, PG1AT, PG1BT, PG1ET-PG1LT, PG1MT-PG1VT, PG1YT-PG3YT, PG5RT-PG6NT, PGA-PGW, PH1AT, PH1FT, PH1UT, PH2ST, PH3YT, PH4AT, PH5GT, PH6GT, PH7FT, PH7UT, PH9AT, PH9ST, PH900, PHA-PHW, PJ2AT, PJ2CT, PJ2HT, PJ3-PJ4, PJ5AN, PJA-PJW, PW, PXA-PXX, QAA-QAW, QBA-QBW, QCA-QCW, QDA-QDW, QW, QXA-QXW, QX2ZT, QX3AT, QX3CT, QX3LT, QX3YT, QX4, S, TAA-TAD, TAW99, TA100, TBA-TJF, TJG10, TJJ, TJL-TJW, TK-TL, TMA-TPX, TQ, U, WXQ, WW20, WW30-WW31, WW40, WW50, WX100-WX105, WX140-WX144, WX7-WX9, XCC00, XFE00, XFN96, XFX00, XFX10, XFX20, XFX97, XJW99, XPX00, XPX04, XPX08, XPX99, XW000, XW1-XW5, XX1AT, XX1BT, XX1CT, XX1DT, XX1XT, XX2AT-XX2DT, XX2XT, XX3AT-XX3DT, XX3XT, XX4-XX7, Y, ZC-ZP, ZS-ZX, ZZ

B) Congestive heart failure and hypertension, no procedure field contains any of the following:

FEA-FEW, FFA00, FFA10-FFA30, FFA96, FFB-FFW, FG-FH, FJA00, FJA96, FJB-FJW, FKA-FKW, FK1BT, FLA00, FLA96, FLB-FLW, FMA-FMW, FN1AT, FN1BT, FN1ST, FN1XT, FN1YT, FNA-FNW, FPA-FPF, FPH-FPW, FQ, FXA00-FXN00, TFN10, TFN99, TFP00, TFP40-TFP59

C) Cellulitis, no procedure field contains any of the following:

AA4WT, AA400, AA5LT, AAA-AAW, AB4AT, AB4BT, AB4CT, AB4DT, AB4FT, AB5, AB6AT, AB6BT, AB6CT, AB6DT, ABA-ABW, ACA-ACW, AD-AW, AXX90, BA3AT, BA4KT, BAA, BB2AT, BB3AT, BB4WT, BBA, BC1LT, BC2-BC4, BCA, BD-BW, CAA-CAW, CB, CCA-CCW, CD-CJ, CKA-CKW, CW, DAA-DAW, DB, DCA-DCW, DD, DEA-DEW, DFA-DFE, DFW99, DG, DHA-DLW, DMA-DPW, DQ-DW, EA, EBA-EBW, EC, EDA-EDW, EEA-EW, EF, EGA-EGW, EHA-EHW, EJ-EK, EL3AT, EL3RT, EL3YT, EL4 ,ELA-ELW, EMA-EMW, EP1AT, EP1LT, EP2AT, EP3AT, ENA-ENW, EW, FA, FBA-FBW, FCA-FCE, FD, FE1AT, FE2, FEA-FEW, FF-FL, FMA-FMW, FN1ST, FN1XT, FNA-FNW, FP-FX, GA2-GA4, GAA-GAW, GBA-GBW, GCA, GD1AT, GD1BT, GD1CT, GD1LT, GD2AT, GD2BT, GD2CT, GD3, GDA-GDW, GE1AT, GE1CT, GE1DT, GE2, GEA-GEW, GW, HA0, HA1AT, HA1DT, HA1MT, HA1ST, HA2-HA5, HAA-HAF, HW, JA1LT, JA2-JA3, JAA-JAW, JB, JCA-JCW, JDA-JDW, JE, JF3, JFA-JFW, JGA-JGW, JHA-JHW, JJ1AT, JJ2-JJ8, JJA-JJW, JK1-JK2, J3KAT, JK3BT, JK3CT, JK3FT, JK3LT, JK3NT, JK3RT, JK4-JK5, JKA-JKW, JL1-JL3, JLA-JLW, JM1AT, JM1LT, JM2, JMA-JMW, JN4LT, JW, JX1LT, JX1RT, JXA, KA2AT, KA3AT, KA3CT, KA3DT, KA3LT, KA4-KA6, KAA-KAW, KBA-KBW, KC1AT, KC2AT, KC3AT, KCA-KCW, KDA-KDW, KE1AT, KE1CT, KE2, KEA-KEW, KF1-KF7, KF8AT, KF8KT, KFA-KFW, KGA-KGW, KH1AT, KH1BT, KH1CT, KH1CT, KH1DT, KH1FT, KH1YT, KKA-KKW, KW, KX, LA1, LAA-LAW, LB1AT, LB1YT, LBA-LBW, LCA-LCW, LDA-LDW, LEA-LEW, LF-LW, LX1LT, MAA-MAW, MBA-MBW, MC-MW, NA0, NA6CT, NA7BT, NA7FT, NA7KT, NA7LT, NA9KT, NAA-NAW, NB1AT,NB1BT, NB1ZT, NB2, NBA-NBW, NCA-NCW, NDA-NDW, NEA-NEW, NFA-NFW, NGA-NGW, NHA-NHW, NJ3LT, NK1AT, NK1CT, NK1DT, NK1LT, NK2-NK3, NK4AT, NK4BT, NK5, NK6AT, NK6BT, NK6CT, NK6DT, NK6KT, NK7AT, NX, PA2ZT, PA3-PA5, PA6AT, PA8KT, PA9KT, PAA-PAW, PB1AT, PB1BT, PB1ST, PB1YT, PBA-PBW, PC2DT, PC2ET, PC5AT, PC5BT, PC5DT, PC5ET, PC5GT, PC5HT, PC5JT, PC5NT, PC5PT, PC5YT, PC6DT, PC6ET, PC6FT, PC7NT, PCA-PCW, PD1AT, PD1YT, PD2DT, PD3, PD4ST, PD5YT, PD6YT, PD7YT, PDA-PDW, PE, PG1AT, PG1BT, PG1ET-PG1LT, PG1MT-PG1VT, PG1YT-PG3YT, PG5RT-PG6NT, PGA-PGW, PH1AT, PH1FT, PH1UT, PH2ST, PH3YT, PH4AT, PH5GT, PH6GT, PH7FT, PH7UT, PH9AT, PH9ST, PH900, PHA-PHW, PJ2AT, PJ2CT, PJ2HT, PJ3-PJ4, PJ5AN, PJA-PJW, PW, PXA-PXX, QAA25, QAB00-QAB05, QAB99, QAC, QAD20, QAE-QAF, QAG10-QAG99, QAJ, QBA25, QBB00-QBB05, QBB99, QBC,

QBD20, QBE, QBG10-QBG99, QBJ, QCA25, QCA30, QCB00-QCB05, QCB99, QCC, QCD20, QCE-QCG, QCJ, QDA25, QDB00-QDB05, QDB99, QDC, QDD20, QDE, QDG10-QDG99, QDJ, QXA25, QXB00-QXB05, QXB99, QXC, QXD20, QXE, QXG10-QXG99, QXJ, QX2ZT, QX3AT, QX3CT, QX3LT, QX3YT, QX4, S, TAA-TAD, TAW99, TA100, TBA-TJF, TJG10, TJJ, TJL-TJW, TK-TL, TMA-TPX, TQA-TQD, TQW00, TQW02, TQW30-40, TQW99, TQX00-TQX10, U, WW20, WW30-WW31, WW40, WW50, WX100-WX105, WX140-WX144, WX7-WX9, XCC00, XFE00, XFN96, XFX00, XFX10, XFX20, XFX97, XJW99, XPX00, XPX04, XPX08, XPX99, XW000, XW1-XW5, XX1AT, XX1BT, XX1CT, XX1DT, XX1XT, XX2AT-XX2DT, XX2XT, XX3AT-XX3DT, XX3XT, XX4-XX7, Y, ZC-ZP, ZS-ZX, ZZ

For peer review only

STROBE 2007 (v4) Statement—Checklist of items that should be included in reports of *cohort studies*

Section/Topic	Item #	Recommendation	Reported on page #
**Title and abstract**	1	(*a*) Indicate the study's design with a commonly used term in the title or the abstract	#1
		(*b*) Provide in the abstract an informative and balanced summary of what was done and what was found	#2-3
**Introduction**			
Background/rationale	2	Explain the scientific background and rationale for the investigation being reported	#4-5
Objectives	3	State specific objectives, including any prespecified hypotheses	#4-5
**Methods**			
Study design	4	Present key elements of study design early in the paper	#5-6
Setting	5	Describe the setting, locations, and relevant dates, including periods of recruitment, exposure, follow-up, and data collection	#5-6
Participants	6	(*a*) Give the eligibility criteria, and the sources and methods of selection of participants. Describe methods of follow-up	#6
		(*b*) For matched studies, give matching criteria and number of exposed and unexposed	NA
Variables	7	Clearly define all outcomes, exposures, predictors, potential confounders, and effect modifiers. Give diagnostic criteria, if applicable	#5-6
Data sources/ measurement	8*	For each variable of interest, give sources of data and details of methods of assessment (measurement). Describe comparability of assessment methods if there is more than one group	*#5-6*
Bias	9	Describe any efforts to address potential sources of bias	#5-6 and discussion section #11-12
Study size	10	Explain how the study size was arrived at	#5-6
Quantitative variables	11	Explain how quantitative variables were handled in the analyses. If applicable, describe which groupings were chosen and why	#5-7
Statistical methods	12	(*a*) Describe all statistical methods, including those used to control for confounding	#6-7
		(*b*) Describe any methods used to examine subgroups and interactions	#6-7
		(*c*) Explain how missing data were addressed	NA
		(*d*) If applicable, explain how loss to follow-up was addressed	NA

For peer review only

		(*e*) Describe any sensitivity analyses	#8
**Results**			
Participants	13*	(a) Report numbers of individuals at each stage of study—eg numbers potentially eligible, examined for eligibility, confirmed eligible, included in the study, completing follow-up, and analysed	#7-8
		(b) Give reasons for non-participation at each stage	NA
		(c) Consider use of a flow diagram	NA
Descriptive data	14*	(a) Give characteristics of study participants (eg demographic, clinical, social) and information on exposures and potential confounders	#7
		(b) Indicate number of participants with missing data for each variable of interest	NA
		(c) Summarise follow-up time (eg, average and total amount)	NA
Outcome data	15*	Report numbers of outcome events or summary measures over time	#7-8
Main results	16	(*a*) Give unadjusted estimates and, if applicable, confounder-adjusted estimates and their precision (eg, 95% confidence interval). Make clear which confounders were adjusted for and why they were included	#figure 1
		(*b*) Report category boundaries when continuous variables were categorized	NA
		(*c*) If relevant, consider translating estimates of relative risk into absolute risk for a meaningful time period	NA
Other analyses	17	Report other analyses done—eg analyses of subgroups and interactions, and sensitivity analyses	#7-11
**Discussion**			
Key results	18	Summarise key results with reference to study objectives	#11-12
**Limitations**			
Interpretation	20	Give a cautious overall interpretation of results considering objectives, limitations, multiplicity of analyses, results from similar studies, and other relevant evidence	#11-12
Generalisability	21	Discuss the generalisability (external validity) of the study results	#11
**Other information**			
Funding	22	Give the source of funding and the role of the funders for the present study and, if applicable, for the original study on which the present article is based	#13

*Give information separately for cases and controls in case-control studies and, if applicable, for exposed and unexposed groups in cohort and cross-sectional studies.

For peer review only

BMJ Open

Regional variation of avoidable hospitalisations in a universal health care system – a register based cohort study from Finland 1996-2013

Journal:	*BMJ Open*
Manuscript ID	bmjopen-2019-029592.R1
Article Type:	Research
Date Submitted by the Author:	21-May-2019
Complete List of Authors:	Manderbacka, Kristiina; National Institute for Health and Welfare (THL), Service System Research<br>Arffman, Martti; National Institute of Health and Welfare, Health and Social Services<br>Satokangas, Markku; Terveyden ja hyvinvoinnin laitos, Service System Research; Helsingin Yliopisto, Network of Academic Health Centres and Department of General Practice and Primary Health Care<br>Keskimaki, Ilmo; National Institute of Health and Welfare, Health and Social Services; Tampereen yliopisto, Dept of Social Sciences
**Primary Subject Heading**:	Health services research
Secondary Subject Heading:	Health policy
Keywords:	STATISTICS & RESEARCH METHODS, PRIMARY CARE, Quality in health care < HEALTH SERVICES ADMINISTRATION & MANAGEMENT

SCHOLARONE™
Manuscripts

Regional variation of avoidable hospitalisations in a universal health care system – a register based cohort study from Finland 1996-2013

Kristiina Manderbacka[1], Martti Arffman[1], Markku Satokangas[1,2], Ilmo Keskimäki[1,3]

[1] Service System Research Unit, National Institute for Health and Welfare, P.O.Box 30, 00271 Helsinki, Finland

[2] Network of Academic Health Centres and Department of General Practice and Primary Health Care, P.O. Box 20, 00014 University of Helsinki, Finland

[3] Dept of Social Sciences, University of Tampere, 33014 Tampere, Finland

Corresponding author

Kristiina Manderbacka

Service System Research Unit, National Institute for Health and Welfare (THL), P.O.Box 30, 00271 Helsinki, Finland, tel +358 29 524 7078 email kristiina.manderbacka@thl.fi

Word count

Abstract 272

Main text 3688

For peer review only

ABSTRACT

Objectives

A persistent finding in research concerning health care and hospital use in Western countries has been regional variation in medical practices. The aim of the current study was to examine trends in regional variation of avoidable hospitalisations, i.e. hospitalisations due to conditions treatable in ambulatory care in Finland in 1996-2013 and the influence of different health care levels on them.

Setting

Use of hospital inpatient care in 1996–2013 among the total population in Finland.

Participants

Altogether 1 931 012 hospital inpatient care episodes among all persons residing in Finland identified from administrative registers in Finland in 1996–2013 and alive in Jan 1, 1996.

Outcome measures

We examined hospitalisations due to avoidable causes including vaccine-preventable hospitalisations, hospitalisations due to complications of chronic conditions and acute conditions treatable in ambulatory care. We calculated annual age-adjusted rates per 10 000 person years. Multilevel models were used for studying time trends in regional variation.

Results

There was a steep decline in avoidable hospitalisation rates during the study period. The decline occurred almost exclusively in hospitalisations due to chronic conditions, which diminished by about 60%. The overall correlation between hospital district intercepts and slopes in time was -0.46 ($p<0.05$) among men and -0.20 (ns) among women. Statistically highly significant diminishing

For peer review only

variation was found in hospitalisations due to chronic conditions among both men (-0.90) and women (-0.91). The variation was mainly distributed to the hospital district level.

Conclusions

The results suggest that chronic conditions are managed better in primary care in the whole country than before. Further research is needed on whether this is the case or whether this has more to do with supply of hospital care.

For peer review only

Strengths and limitations of this study

- Our register based data covers the total population in Finland identified from register sources in Finland over a 18-year follow-up period.
- We used two indicators of region of residence that are directly connected to the organisation of public health care for the residents enabling us to disentangle the association of each organisational level to regional variations in hospitalisation rates.
- Multilevel models enabled us to capture the hierarchical nature of the data (health centres nested in hospital districts) allowing us to analyse the association more efficiently.
- We were not able to control for need for care i.e. disease prevalence or severity, and thus cannot estimate what part of regional differences in avoidable hospitalisations would be warranted due to differences in disease prevalence and/or severity.

For peer review only - http://bmjopen.bmj.com/site/about/guidelines.xhtml

BACKGROUND OF THE STUDY

A persistent finding in research concerning health care and hospital use in Western countries has been regional variation in medical practices. While there are regional variations within countries in disease prevalence, earlier studies have suggested that at least some of the variation in medical care is unwarranted, i.e. within country variation does not disappear when need is accounted for [1]. There is also a need to examine whether medical practice variation changes in time [1]. In a relatively recent systematic review Corallo et al [2] report a large number of studies examining medical practice variation from OECD countries covering regional differences in ambulatory care, medical admissions, elective surgery, as well as emergency hospitalisations and avoidable hospitalisations, i.e. hospitalisations due to conditions treatable in ambulatory care. Avoidable hospitalisations are commonly used as an indirect indicator of access to and quality of health care [3,4]. Earlier studies use slightly different lists of conditions, but generally three types of conditions are examined: admissions that should be avoided with timely access to preventive measures, ambulatory treatment of acute conditions and managing of certain chronic disease in an ambulatory setting.

While there are earlier studies examining regional differences in avoidable hospitalisations, many examine only some parts of the country in question [5-10], specific age groups [5,7,10], are cross-sectional [5,9,11,12] and/or study these hospitalisations in specific patient groups, e.g. persons with diabetes [13]. Further, some countries publish regularly Atlases of regional differences in medical practices, some of which also report avoidable hospitalisations; see e.g. [14,15].

The primary aim of this register-based study was to examine trends in avoidable hospitalisations in 1996-2013, whether regional variation increased or decreased in relation to the national trend i.e.,

For peer review only - http://bmjopen.bmj.com/site/about/guidelines.xhtml

possible increase or decrease in regional variation and, secondarily, if so, on what level of health care the variation occurred. Few earlier studies have reported development of geographic variation in avoidable hospitalisations with a longitudinal setting over an extensive time period. The Finnish health-care system provides a good case for examining avoidable hospitalisations as the system is universal in coverage and therefore, in general, supports equal access to both primary and specialised health-care according to need [16]. The system is mainly financed by tax revenues and user-fees are generally low. The system also supports evidence based care as there are accepted guidelines for the treatment of altogether 106 conditions in the National Current Care guidelines system [17] . There are ca 160 health centres providing ambulatory primary care in Finland, owned and managed by municipalities themselves or in co-operation with other municipalities. They are the primary producers of ambulatory services for the residents. However, there have been difficulties in access in some areas. There are 21 hospital districts in Finland providing specialist services to the residents. They are owned, funded and managed by federations of municipalities.

MATERIALS AND METHODS

The data

We obtained avoidable hospitalisations for the total Finnish population aged 20 years or older in 1996-2013 from the Finnish hospital discharge register, maintained by the National Institute for Health and Welfare (THL). The linkages were performed by competent authorities and the research group received pseudonymized data. Ethical approval for the study was received from the Research Ethics Committee of THL (decision #7/2013 §586).

For peer review only - http://bmjopen.bmj.com/site/about/guidelines.xhtml

We used the UK definition of avoidable hospitalisations [3,4] with an inclusion of ICD10 codes J18.9 and J09 (pneumonia). Avoidable hospitalisations were classified into three subcategories: acute, chronic, and vaccine-preventable (for diagnoses included and their ICD10-codes, see appendix 1). Hospitalisations were combined if they occurred within one day from each other. Data on hospitalisations were linked into demographic data obtained from Statistics Finland.

We used two indicators of region of residence: (1) health centre area (single municipality or co-operation area of municipalities) that is responsible for organising primary health care for the residents and (2) hospital district (owned by the federations of municipalities), which is responsible for organising the public specialist services in the area. Publicly funded hospitals produce ca 95% of all specialised inpatient services in Finland. The municipality of residence was linked for each individual at the end of the year preceding hospitalisation. The small autonomous region of Åland was excluded from the analyses. Age at hospitalisation was classified into 5-year age brackets with those 85 years or older as the oldest group. Those living permanently in institutions were excluded from analyses. Total Finnish population was used as the population at risk in the analyses. All analyses were conducted separately for men and women.

Statistical methods

We calculated national age-standardised rates for each category of avoidable hospitalisations per 10 000 person years with direct standardisation method with the European standard population as standard population. We calculated autocorrelations in each of the categories both in the health centre and in the hospital district level to study consistency in hospitalisation rates and their suitability for further analysis. We found autocorrelations in health centre level vary a lot suggesting that this level was not suitable for the analysis as the result suggests inconsistency in the development in time. The hospital district level autocorrelations were relatively low among

For peer review only - http://bmjopen.bmj.com/site/about/guidelines.xhtml

men in vaccine-preventable hospitalisations, averaging 0.22, among women the autocorrelations were higher, averaging 0.35. In hospitalisations due to acute conditions, the results were rather similar with autocorrelations averaging 0.44 among men and 0.36 among women. The autocorrelations for hospitalisations due to chronic conditions were higher, with average of 0.78 among both men and women. These numbers suggest that hospital district patterns in hospitalisations were relatively consistent in time and allowed us to examine possible convergence of hospitalisation rates, that is, decrease in the variation, at the hospital district level.

Next, we performed two-level models to receive annual z-scores of the age-standardised hospitalisation rates in each hospital district and category adjusted for polynomial function of time with normally distributed random intercept and slope terms at the hospital district level. Z-scores were then used to examine the hospitalization rates from different categories of avoidable hospitalisations in a comparable scale. Multilevel models were applied to manage the hierarchical nature of the data and polynomial function of time to capture the general national trend in each category. We calculated correlation between the random intercept and slope terms in these models to obtain an estimate of convergence in hospitalisation rates. A statistically significant negative correlation between random intercepts and random slopes was interpreted as a decrease of regional variation in time. In that case high random intercepts were correlated with large negative random slopes, and vice versa, indicating hospital districts approaching the average.

A similar procedure was fitted to all hospitalisation rates due to avoidable causes simultaneously where polynomial function of time was adjusted for each category of avoidable hospitalisations to calculate an estimate for possible general convergence of hospitalisation rates between regions, i.e. whether the regional variation in rates decreased more or less in relation to the decreasing national average [1,18].

For peer review only

In a separate analysis, we used three-level Poisson regression models to examine whether variation between avoidable hospitalisations occurred mainly in the hospital district or health centre level. In these models, year was controlled as a continuous variable and age group as a categorical variable with hospital districts and health centres as random variables.

In order to assess whether there were changes in the levels of health care where the regional variation occurred, we estimated models separately for the years 1996−1999 and 2010−2013. Wald test was used to assess the statistical significance of random components in the models.

Also, we calculated intraclass correlation coefficients (ICC) for the two levels [19].Variation in age-adjusted avoidable hospitalization rates in health centre areas was illustrated with scatter plots in the last four years of study period. The extremal quotient (EQ) was defined as the ratio of the largest and smallest rates.

Patient and public involvement

As the study was based on pseudonymized data from administrative registers, patients were not involved in the study. The results will be disseminated to the public through summary articles after the original studies have been published.

RESULTS

There were altogether 983 966 avoidable hospital admissions among men and 947 046 among women during the study period. More than half of the hospitalisations were due to chronic conditions.

[Figure 1 around here]

For peer review only - http://bmjopen.bmj.com/site/about/guidelines.xhtml

For peer review only

Figure 1 presents the national rates of avoidable hospitalisations. Avoidable hospitalisations decreased consistently during the study period. The total rate of avoidable hospitalisations was 535 per 10 000 person years (py) among men in 1996 and 306 per 10 000 py in 2013. Among women the rates were 340 in 1996 and 221 in 2013. The rates were larger among men throughout the study period in each of the avoidable hospitalisation categories. While the rates remained relatively stable in hospitalisations due to vaccine-preventable conditions and acute conditions, there was a large decrease in hospitalisations for chronic conditions both among men and among women during the study period.

Table 1. Multilevel model based correlation between hospital district intercepts and slopes in time between 1996 and 2013

	Men	Women
Preventable	-0.02 ns	0.44 ns
Chronic conditions	-0.90***	-0.91***
Acute conditions	-0.45*	0.01 ns

* p<0.05, **p<0.01, *** p<0.001, ns statistically non-significant

In multilevel analysis (Table 1), the correlation between hospital district intercepts and slopes in time was negative among men suggesting decreasing variation when all categories of avoidable hospitalisations were examined together. Among women, no such correlation was found. The correlations were -.0.46 (p<0.05) among men and -0.22 (ns) among women. However, when examining categories of avoidable hospitalisation separately, negative correlations indicating

For peer review only - http://bmjopen.bmj.com/site/about/guidelines.xhtml

diminishing variation of hospitalisation rates in hospital districts were found in rates due to chronic conditions among both genders and in rates due to acute conditions among men. In other categories, no correlations indicating change in regional variation were found. The results were mostly similar when health centre level was used in the analysis (data not shown).

Finally we examined to which regional level (health centre level vs. hospital district level) the variation was mainly distributed in each of the categories of avoidable hospitalisations in the beginning (1996–1999) and in the end (2010–2013) of the study period. We examined these two periods due to decrease in avoidable hospitalisation rates during the study period (Table 2).

Table 2. The distribution of variance in avoidable hospitalisations to health centre (HC) and hospital district (HD) level in Finland in 1996-1999 and 2010-2013 (Poisson multilevel models controlling for year as a continuous variable and age as a categorical variable; estimates of variance components and their intraclass correlations (ICC))

			1996-1999		2010-2013	
	Category	Level	Estimate	ICC	Estimate	ICC
Men	Preventable	HD	0.024**	0.022	0.027**	0.024
		HC	0.022***	0.021	0.024***	0.023
	Chronic	HD	0.051**	0.046	0.064**	0.056
		HC	0.019***	0.012	0.027***	0.025
	Acute	HD	0.025**	0.022	0.039**	0.034
		HC	0.015***	0.014	0.024***	0.023
Women	Preventable	HD	0.032**	0.028	0.033**	0.029
		HC	0.033***	0.031	0.029***	0.027
	Chronic	HD	0.054**	0.047	0.059**	0.051
		HC	0.031***	0.029	0.033***	0.030
	Acute	HD	0.026**	0.024	0.043**	0.038
		HC	0.019***	0.018	0.029***	0.027

*p<0.05, ** p<0.01, *** p<0.001

In hospitalisations due to vaccine-preventable conditions the variance was equally distributed to both levels in the late 1990s and remained similar at the end of the study period among both men and women. In hospitalisations due to chronic conditions the variance was distributed mainly to the hospital district level. Similar, but smaller variation was found in hospitalisations due to acute

For peer review only - http://bmjopen.bmj.com/site/about/guidelines.xhtml

conditions. Health centre level variance was also statistically significant in all categories of avoidable hospitalisations among both men and women. However, proportions of variance explained by the two levels remained relatively low according to ICCs.

[Figure 2 around here]

For peer review only

Figure 2 illustrates health centre level variation in age-adjusted rates in the three categories of avoidable hospitalisations in the end of the study period (2010-2013). In the figure, each dot represents one health centre. The variation was, in general, smaller among women compared to men in each of the categories. In vaccine-preventable hospitalisations the health centre rates were close to each other with some outliers especially towards the higher end. Extremal quotient (EQ) among men was 4.80 and among women 4.58. In hospitalisations due to acute conditions, the variation was similar but smaller with an EQ of 4.31 among men and 4.50 among women. In hospitalisations due to chronic conditions the variation was large, with and EQ of 4.81 for men and 5.36 for women.

DISCUSSION

Overview of results

We found a decreasing trend in avoidable hospitalisations from the mid-1990s to the early 2010s. This trend was clear in avoidable hospitalisations due to chronic conditions, but not in vaccine-preventable hospitalisations or hospitalisations due to acute conditions. There were large hospital district variations in avoidable hospitalisations due to chronic conditions. Also, in multilevel analyses the correlation between hospital district intercepts and slopes was negative in these

For peer review only - http://bmjopen.bmj.com/site/about/guidelines.xhtml

hospitalisations, indicating decreasing hospital district variation and convergence of rates between hospital districts during the study period. Our result concerning relatively large regional variation in avoidable hospitalisations is in line with earlier results from other countries [5-9,11,20, 21] and in different categories of avoidable hospitalisations [14,15]. Several reasons have been presented to regional variation, including differences in disease prevalence, rurality, age structure and socioeconomic composition between regions [6-9,22,23], as well as access to and accessibility of primary health care and supply of hospital beds [9,10,20,21].

Earlier studies have mainly been cross-sectional and have not examined trends in avoidable hospitalisations, but our result concerning decreasing variation is in line with results from Canada [11]. Our study did not include data on disease prevalence or change in it. However, an earlier Finnish study examining persons with diabetes found a similar decrease in hospitalisations due to complications and convergence of hospitalisation rates in the early 2000s suggesting better control of diabetes in primary care [13]. It is possible that disease management improved in other conditions as well thus decreasing the need for specialist hospital care. Further, current care guidelines together with disease specific programmes may have resulted in more uniform disease management in primary care thus decreasing regional differences in hospitalisations.

Further research is needed to study the effects of differences in the regional sociodemographic composition of the population and supply side factors to avoidable hospitalisations. On the supply side, an important question is whether the variation is driven by access to and accessibility of primary care or supply of hospital inpatient care. Our results on larger variation in hospital district level in hospitalisations due to chronic conditions support previous results [6,24], which suggest that different categories of avoidable hospitalisations should be analysed separately.

For peer review only - http://bmjopen.bmj.com/site/about/guidelines.xhtml

Methodological considerations

A major strength of our study was that we were able to use individual level data of the total population of Finland over a period of 18 years. The data concerning avoidable hospitalisations were derived from the Finnish Hospital Discharge Register, which has been found to have, in general, good quality and coverage [25]. Use of multilevel models enabled us to capture the hierarchical nature of the data (health centres nested within hospital districts) and the statistical approach allowed us to analyse the development of regional variation in an efficient way. However, even this modern statistical approach is limited in modelling complex phenomenon with considerable individual variation as represented by small ICCs.

Though earlier studies have shown that avoidable hospitalisations associate to factors like need for care i.e. disease prevalence or severity, we were unable to control for them. Thus we cannot directly evaluate what part of regional differences in avoidable hospitalisations would be warranted due to differences in disease prevalence and/or severity. It is still unclear whether the decrease in rates of the chronic disease category and their regional variation occurred mainly due to the development of primary care or whether it is due to overall reduction in hospital beds. This requires further studies assessing both the development of supply of hospital beds and number of total hospitalisations.

Nor could we evaluate whether primary care has an effect on these differences, which has been strongly debated. Our results of systematically developing regional variation – and its over time division between primary care providers and hospital districts – lay a foundation for further analysis. We consider that analysing how the fore mentioned factors simultaneously affect the variation of several regional levels and over-time annual trends, poses methodological challenges. This necessitates an independent study with an approach that is optimized to test for these

For peer review only

For peer review only - http://bmjopen.bmj.com/site/about/guidelines.xhtml

associations. Future studies need also to take into account variation in sociodemographic composition of regions. Our study controlled for gender and age, but not for different dimensions of socioeconomic position.

Conclusions

Large but decreasing regional variation was found in our study in avoidable hospitalisations due to chronic conditions. These hospitalisations occur mainly due to complications of the underlying disease. The result may suggest that chronic conditions are better managed in primary care in the whole country than before. Further research is needed on whether this is the case or whether this has more to do with supply of hospital care.

For peer review only

For peer review only - http://bmjopen.bmj.com/site/about/guidelines.xhtml

Funding

The study was funded by the Strategic Research Council (grants 312 703 and 312 708) and the Medical Research Council (grant 277 939) at the Academy of Finland, but the Academy was not involved in study design, data collection, findings or decision to publish.

Authors' contributions

KM contributed to the conception and design of the study, planning of analyses, and drafted the manuscript. MA contributed to the conception and design of the study, performed the statistical analyses and took part in the revision of the manuscript for important intellectual content. MS contributed to the conception and design of the study, planning of analyses, and took part in the revision of the manuscript for important intellectual content. IK contributed to the conception and design of the study, planning of analyses and took part in the revision of the manuscript for important intellectual content. All authors have read and approved the final manuscript.

Competing interests

The authors declare no conflicts of interest that could have biased the work.

Data sharing statement

Due to Finnish data protection legislation, no further data is available.

For peer review only

Bibliography

1 Westert GP, Groenewegen PP, Boshuizen HC, et al. Medical practice variations in hospital care; time trends of a spatial phenomenon. *Health Place* 2004;**10**:215-220.

2 Corallo AN, Croxford R, Goodman DC, et al. A systematic review of medical practice variation in OECD countries. *Health Policy* 2014;**114**:5-14.

3 Purdy S, Griffin T, Salisbury C, et al. Ambulatory care sensitive conditions: terminology and disease coding need to be more specific to aid policy makers and clinicians. *Public Health* 2009;**123**:169-173.

4 Tian Y, Dixon A, Gao H. Data briefing: Emergency hospital admissions for ambulatory care-sensitive conditions: identifying the potential for reductions. London: *The Kings Fund* 2012.

5 Magan P, Otero A, Alberquilla A, et al. Geographic variations in avoidable hospitalizations in the elderly, in a health system with universal coverage. *BMC Health Serv Res* 2008;**8**:42.

6 Falster MO, Jorm LR, Douglas KA, et al. Sociodemographic and health characteristics, rather than primary care supply, are major drivers of geographic variation in preventable hospitalizations in Australia. *Med Care* 2015;**53**:436-445.

7 Hale N, Probst J, Robertson A. Rural Area Deprivation and Hospitalizations Among Children for Ambulatory Care Sensitive Conditions. *J Community Health* 2016;**41**:451-460.

8 Gusmano MK, Weisz D, Rodwin VG, et al. Disparities in access to health care in three French regions. *Health Policy* 2014;**114**:31-40.

For peer review only

For peer review only - http://bmjopen.bmj.com/site/about/guidelines.xhtml

9 Probst JC, Laditka JN, Laditka SB. Association between community health center and rural health clinic presence and county-level hospitalization rates for ambulatory care sensitive conditions: an analysis across eight US states. *BMC Health Serv Res* 2009;**9**:134.

10 Butler DC, Thurecht L, Brown L, et al. Social exclusion, deprivation and child health: a spatial analysis of ambulatory care sensitive conditions in children aged 0-4 years in Victoria, Australia. *Soc Sci Med* 2013;**94**:9-16.

11 Sanchez M, Vellanky S, Herring J, et al. Variations in Canadian rates of hospitalization for ambulatory care sensitive conditions. *Healthc Q* 2008;**11**:20-22.

12 Busby J, Purdy S, Hollingworth W. Opportunities for primary care to reduce hospital admissions: a cross-sectional study of geographical variation. *Br J Gen Pract* 2016:e20-e28.

13 Manderbacka K, Arffman M, Lumme S, et al. Regional trends in avoidable hospitalisations due to complications among population with diabetes in Finland in 1996-2011: a register-based cohort study. *BMJ Open* 2016;**6**:011620.

14 Australian Commission of Quality and Safety in Health Care. The Second Australian Atlas of Healthcare Variation. Sydney: *Australian Commission on Quality and Safety in Health Care* 2017.

15 NHS Rightcare. NHS Atlas of Variation in Healthcare 2015. Leeds: *NHS Rightcare* 2015.

16 Vuorenkoski L, Mladovsky P, Mossialos E. Finland: Health system review. *Health Systems in Transition* 2008;**10(4)**:168.

17 The Finnish Medical Society Duodecim. Current Care Guidelines. 2018. https://www.duodecim.fi/english/products/current-care-guidelines/ (accessed 11.11.2018).

For peer review only

18 Snijders T. Analysis of longitudinal data using the hierarchical linear model. *Quality and quantity* 1995;**30**:405-426.

19. Nakagawa S, Johnson PCD,Schielzeth H. 2017 The coefficient of determination R2 and intra-class correlation coefficient from generalized linear mixed effects models revisited and expanded. *J R Soc Interface* 2017; **14**: 0213. http://dx.doi.org/10.1098/rsif.2017.0213

20 Sexton E, Bedford D. GP supply, deprivation and emergency admission to hospital for COPD and diabetes complications in counties across Ireland: an exploratory analysis. *Ir J Med Sci* 2016;**185**:453-461.

21 Weeks WB, Ventelou B, Paraponaris A. Rates of admission for ambulatory care sensitive conditions in France in 2009-2010: trends, geographic variation, costs, and an international comparison. *Eur J Health Econ* 2016;**17**:453–470.

22 Löfqvist T, Burström B, Walander A, et al. Inequalities in avoidable hospitalisation by area income and the role of individual characteristics: a population-based register study in Stockholm County, Sweden. 2013 Sep 30. *BMJ Qual Saf* 2013;**Sep 30**:001715. [Epub ahead of print].

23 Ansari Z, Haider SI, Ansari H, et al. Patient characteristics associated with hospitalisations for ambulatory care sensitive conditions in Victoria, Australia. *BMC Health Serv Res* 2012;**12**:475.

24 Saver BG, Wang CY, Dobie SA, et al. The central role of comorbidity in predicting ambulatory care sensitive hospitalizations. *Eur J Public Health* 2014;**24**:66-72.

25 Sund R. Quality of the Finnish Hospital Discharge Register: a systematic review. *Scan J Public Health* 2012;**40**:505-515.

For peer review only - http://bmjopen.bmj.com/site/about/guidelines.xhtml

Figure legends

Figure 1. Trends in avoidable hospitalisations among men and women by category in Finland 1996-2013, age-standardised rates per 10 000 person years

Figure 2. Health centre level variation in avoidable hospitalisations among men and women in 2010-2013, age-adjusted rates per 10 000 person years

For peer review only

For peer review only - http://bmjopen.bmj.com/site/about/guidelines.xhtml

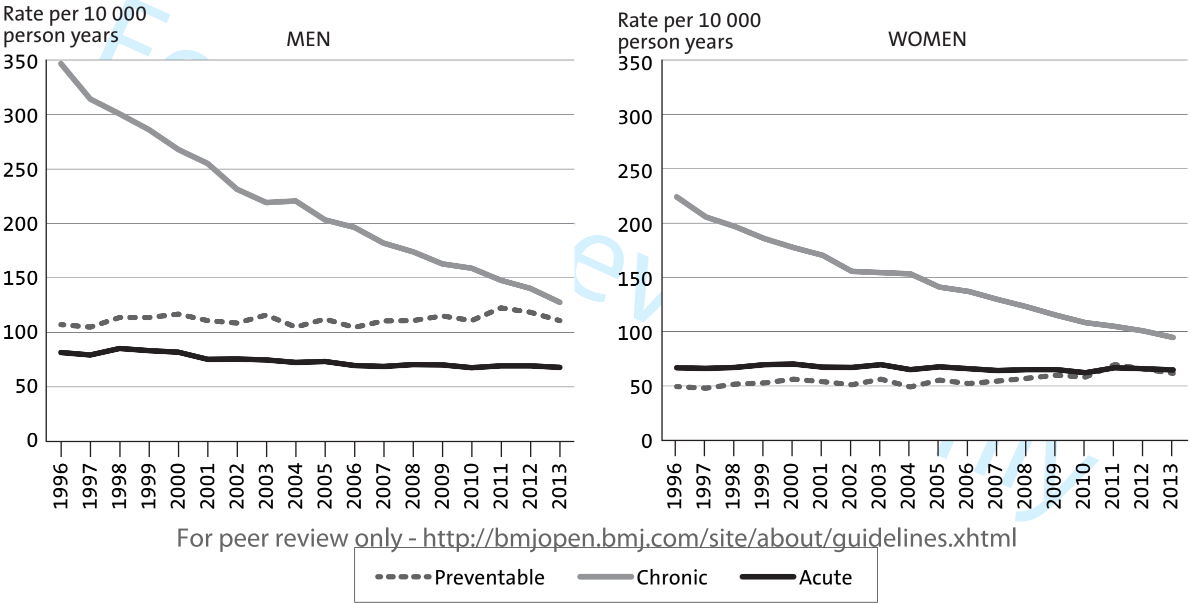

For peer review only - http://bmjopen.bmj.com/site/about/guidelines.xhtml

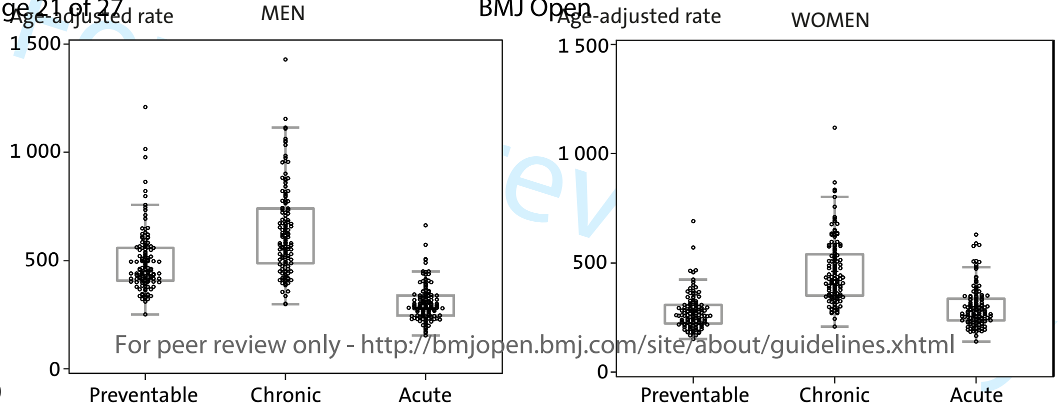

For peer review only - http://bmjopen.bmj.com/site/about/guidelines.xhtml

Appendix 1. Conditions included in each category of avoidable hospitalisations

Condition	ICD-9 (Finnish version)	ICD-10	Definition notes
**Vaccine-preventable**			
Bacterial Pneumonia & Influenza	481, 4822A, 4823A, 4824A, 4829X, 483, 485, 487	J09, J10, J11, J13, J14, J15.3, J15.4, J15.7, J15.9, J16.8, J18.1, J18.8, J18.9	In any diagnosis field, do not accept if D57 is as a secondary diagnose
Immunization-Related and Preventable Conditions	032, 033, 037, 045, 055, 056, 0703A, 072, 3200A	A35, A36, A37, A80, B05, B06, B16.1, B16.9, B18.0, B18.1, B26, G00.0, M01.4	In any diagnosis field
**Chronic**			
Acute Bronchitis	4660A	J20	Only when primary diagnose, and J41-J44 or J47 as a secondary diagnose
Angina	4110, 4111, 413	I20, I24.0, I24.8, I24.9	Primary diagnose only, exclude cases with surgical procedures (A)
Asthma	493	J45, J46	Primary diagnose only
Chronic Obstructive Pulmonary Disease (COPD)	491, 492, 494, 496	J41, J42, J43, J44, J47	Primary diagnose only
Congestive Heart Failure	4029B, 428	I11.0, I50, J81	Primary diagnose only, exclude cases with cardiac procedures (B)
Diabetes Complications	2501-2508	E10.0-10.8, E11.0-E11.8, E12.0-E12.8, E13.0-E13.8, E14.0-E14.8	Primary diagnose only
Hypertension	401, 4029A	I10, I11.9	Primary diagnose only, exclude cases with cardiac procedures (B)
Iron Deficiency Anaemia	280	D50.1-D50.9	Primary diagnose only
Nutritional Deficiencies	260, 261, 262, 2680A, 2681A	E40, E41, E42, E43, E55.0, E64.3	Primary diagnose only
**Acute**			
Cellulitis	681, 682, 683, 6860A-6868C	L03, L04, L08.0, L08.8, L88, L98.0	Primary diagnose only, exclude cases with other surgical procedures than skin procedures (C)
Convulsions and Epilepsy	345, 6426, 7803A	G40, G41, O15, R56	Primary diagnose only
Dehydration and Gastroenteritis	2765A, 5583A, 5589X	E86, K52.2, K52.8, K52.9	Primary diagnose only
Dental Conditions	101, 521, 522, 523, 525, 528	A69.0, K02-K06, K08, K09.8, K09.9, K12, K13	Primary diagnose only
Gangrene	7854A+2506	R02	In any diagnosis field
Kidney and Urinary Tract Infections	5900A, 5901A	N10, N11, N12, N13.6	Primary diagnose only
Pelvic Inflammatory Disease	614	N70, N73, N74	Primary diagnose only
Perforated or Bleeding Ulcer	5310B-5310C, 5311B-5311C, 5312B-5312C, 5313B-5313C, 5314B-5314C, 5315B-5315C, 5320B-5230C, 5321B-5321C, 5322B-5322C, 5323B-5323C, 5324B-5324C, 5325B-5325C, 5340B-5340C, 5341B-5341C, 5348B-5348C	K25.0-K25.2, K25.4-K25.6, K26.0-K26.2, K26.4-K26.6, K27.0-K27.2, K27.4-K27.6, K28.0-K28.2, K28.4-K28.6	Primary diagnose only

For peer review only

Severe ENT infection	0340, 382, 462, 463, 465, 4721A	H66, H67, J02, J03, J06, J31.2	Primary diagnose only

For peer review only

A) Angina, no procedure field contains any of the following (NCSP, Nordic Classification of Surgical Procedures):

AA4WT, AA400, AA5LT, AAA-AAW, AB4AT, AB4BT, AB4CT, AB4DT, AB4FT, AB5, AB6AT, AB6BT, AB6CT, AB6DT, ABA-ABW, ACA-ACW, AD-AW, AXX90, BA3AT, BA4KT, BAA, BB2AT, BB3AT, BB4WT, BBA, BC1LT, BC2-BC4, BCA, BD-BW, CAA-CAW, CB, CCA-CCW, CD-CJ, CKA-CKW, CW, DAA-DAW, DB, DCA-DCW, DD, DEA-DEW, DFA-DFE, DFW99, DG, DHA-DLW, DMA-DPW, DQ-DW, EA, EBA-EBW, EC, EDA-EDW, EEA-EW, EF, EGA-EGW, EHA-EHW, EJ-EK, EL3AT, EL3RT, EL3YT, EL4 ,ELA-ELW, EMA-EMW, EP1AT, EP1LT, EP2AT, EP3AT, ENA-ENW, EW, FA, FBA-FBW, FCA-FCE, FD, FE1AT, FE2, FEA-FEW, FF-FL, FMA-FMW, FN1ST, FN1XT, FNA-FNW, FP-FX, GA2-GA4, GAA-GAW, GBA-GBW, GCA, GD1AT, GD1BT, GD1CT, GD1LT, GD2AT, GD2BT, GD2CT, GD3, GDA-GDW, GE1AT, GE1CT, GE1DT, GE2, GEA-GEW, GW, HA0, HA1AT, HA1DT, HA1MT, HA1ST, HA2-HA5, HAA-HAF, HW, JA1LT, JA2-JA3, JAA-JAW, JB, JCA-JCW, JDA-JDW, JE, JF3, JFA-JFW, JGA-JGW, JHA-JHW, JJ1AT, JJ2-JJ8, JJA-JJW, JK1-JK2, J3KAT, JK3BT, JK3CT, JK3FT, JK3LT, JK3NT, JK3RT, JK4-JK5, JKA-JKW, JL1-JL3, JLA-JLW, JM1AT, JM1LT, JM2, JMA-JMW, JN4LT, JW, JX1LT, JX1RT, JXA, KA2AT, KA3AT, KA3CT, KA3DT, KA3LT, KA4-KA6, KAA-KAW, KBA-KBW, KC1AT, KC2AT, KC3AT, KCA-KCW, KDA-KDW, KE1AT, KE1CT, KE2, KEA-KEW, KF1-KF7, KF8AT, KF8KT, KFA-KFW, KGA-KGW, KH1AT, KH1BT, KH1CT, KH1CT, KH1DT, KH1FT, KH1YT, KKA-KKW, KW, KX, LA1, LAA-LAW, LB1AT, LB1YT, LBA-LBW, LCA-LCW, LDA-LDW, LEA-LEW, LF-LW, LX1LT, MAA-MAW, MBA-MBW, MC-MW, NA0, NA6CT, NA7BT, NA7FT, NA7KT, NA7LT, NA9KT, NAA-NAW, NB1AT,NB1BT, NB1ZT, NB2, NBA-NBW, NCA-NCW, NDA-NDW, NEA-NEW, NFA-NFW, NGA-NGW, NHA-NHW, NJ3LT, NK1AT, NK1CT, NK1DT, NK1LT, NK2-NK3, NK4AT, NK4BT, NK5, NK6AT, NK6BT, NK6CT, NK6DT, NK6KT, NK7AT, NX, PA2ZT, PA3-PA5, PA6AT, PA8KT, PA9KT, PAA-PAW, PB1AT, PB1BT, PB1ST, PB1YT, PBA-PBW, PC2DT, PC2ET, PC5AT, PC5BT, PC5DT, PC5ET, PC5GT, PC5HT, PC5JT, PC5NT, PC5PT, PC5YT, PC6DT, PC6ET, PC6FT, PC7NT, PCA-PCW, PD1AT, PD1YT, PD2DT, PD3, PD4ST, PD5YT, PD6YT, PD7YT, PDA-PDW, PE, PG1AT, PG1BT, PG1ET-PG1LT, PG1MT-PG1VT, PG1YT-PG3YT, PG5RT-PG6NT, PGA-PGW, PH1AT, PH1FT, PH1UT, PH2ST, PH3YT, PH4AT, PH5GT, PH6GT, PH7FT, PH7UT, PH9AT, PH9ST, PH900, PHA-PHW, PJ2AT, PJ2CT, PJ2HT, PJ3-PJ4, PJ5AN, PJA-PJW, PW, PXA-PXX, QAA-QAW, QBA-QBW, QCA-QCW, QDA-QDW, QW, QXA-QXW, QX2ZT, QX3AT, QX3CT, QX3LT, QX3YT, QX4, S, TAA-TAD, TAW99, TA100, TBA-TJF, TJG10, TJJ, TJL-TJW, TK-TL, TMA-TPX, TQ, U, WXQ, WW20, WW30-WW31, WW40, WW50, WX100-WX105, WX140-WX144, WX7-WX9, XCC00, XFE00, XFN96, XFX00, XFX10, XFX20, XFX97, XJW99, XPX00, XPX04, XPX08, XPX99, XW000, XW1-XW5, XX1AT, XX1BT, XX1CT, XX1DT, XX1XT, XX2AT-XX2DT, XX2XT, XX3AT-XX3DT, XX3XT, XX4-XX7, Y, ZC-ZP, ZS-ZX, ZZ

B) Congestive heart failure and hypertension, no procedure field contains any of the following:

FEA-FEW, FFA00, FFA10-FFA30, FFA96, FFB-FFW, FG-FH, FJA00, FJA96, FJB-FJW, FKA-FKW, FK1BT, FLA00, FLA96, FLB-FLW, FMA-FMW, FN1AT, FN1BT, FN1ST, FN1XT, FN1YT, FNA-FNW, FPA-FPF, FPH-FPW, FQ, FXA00-FXN00, TFN10, TFN99, TFP00, TFP40-TFP59

C) Cellulitis, no procedure field contains any of the following:

AA4WT, AA400, AA5LT, AAA-AAW, AB4AT, AB4BT, AB4CT, AB4DT, AB4FT, AB5, AB6AT, AB6BT, AB6CT, AB6DT, ABA-ABW, ACA-ACW, AD-AW, AXX90, BA3AT, BA4KT, BAA, BB2AT, BB3AT, BB4WT, BBA, BC1LT, BC2-BC4, BCA, BD-BW, CAA-CAW, CB, CCA-CCW, CD-CJ, CKA-CKW, CW, DAA-DAW, DB, DCA-DCW, DD, DEA-DEW, DFA-DFE, DFW99, DG, DHA-DLW, DMA-DPW, DQ-DW, EA, EBA-EBW, EC, EDA-EDW, EEA-EW, EF, EGA-EGW, EHA-EHW, EJ-EK, EL3AT, EL3RT, EL3YT, EL4 ,ELA-ELW, EMA-EMW, EP1AT, EP1LT, EP2AT, EP3AT, ENA-ENW, EW, FA, FBA-FBW, FCA-FCE, FD, FE1AT, FE2, FEA-FEW, FF-FL, FMA-FMW, FN1ST, FN1XT, FNA-FNW, FP-FX, GA2-GA4, GAA-GAW, GBA-GBW, GCA, GD1AT, GD1BT, GD1CT, GD1LT, GD2AT, GD2BT, GD2CT, GD3, GDA-GDW, GE1AT, GE1CT, GE1DT, GE2, GEA-GEW, GW, HA0, HA1AT, HA1DT, HA1MT, HA1ST, HA2-HA5, HAA-HAF, HW, JA1LT, JA2-JA3, JAA-JAW, JB, JCA-JCW, JDA-JDW, JE, JF3, JFA-JFW, JGA-JGW, JHA-JHW, JJ1AT, JJ2-JJ8, JJA-JJW, JK1-JK2, J3KAT, JK3BT, JK3CT, JK3FT, JK3LT, JK3NT, JK3RT, JK4-JK5, JKA-JKW, JL1-JL3, JLA-JLW, JM1AT, JM1LT, JM2, JMA-JMW, JN4LT, JW, JX1LT, JX1RT, JXA, KA2AT, KA3AT, KA3CT, KA3DT, KA3LT, KA4-KA6, KAA-KAW, KBA-KBW, KC1AT, KC2AT, KC3AT, KCA-KCW, KDA-KDW, KE1AT, KE1CT, KE2, KEA-KEW, KF1-KF7, KF8AT, KF8KT, KFA-KFW, KGA-KGW, KH1AT, KH1BT, KH1CT, KH1CT, KH1DT, KH1FT, KH1YT, KKA-KKW, KW, KX, LA1, LAA-LAW, LB1AT, LB1YT, LBA-LBW, LCA-LCW, LDA-LDW, LEA-LEW, LF-LW, LX1LT, MAA-MAW, MBA-MBW, MC-MW, NA0, NA6CT, NA7BT, NA7FT, NA7KT, NA7LT, NA9KT, NAA-NAW, NB1AT,NB1BT, NB1ZT, NB2, NBA-NBW, NCA-NCW, NDA-NDW, NEA-NEW, NFA-NFW, NGA-NGW, NHA-NHW, NJ3LT, NK1AT, NK1CT, NK1DT, NK1LT, NK2-NK3, NK4AT, NK4BT, NK5, NK6AT, NK6BT, NK6CT, NK6DT, NK6KT, NK7AT, NX, PA2ZT, PA3-PA5, PA6AT, PA8KT, PA9KT, PAA-PAW, PB1AT, PB1BT, PB1ST, PB1YT, PBA-PBW, PC2DT, PC2ET, PC5AT, PC5BT, PC5DT, PC5ET, PC5GT, PC5HT, PC5JT, PC5NT, PC5PT, PC5YT, PC6DT, PC6ET, PC6FT, PC7NT, PCA-PCW, PD1AT, PD1YT, PD2DT, PD3, PD4ST, PD5YT, PD6YT, PD7YT, PDA-PDW, PE, PG1AT, PG1BT, PG1ET-PG1LT, PG1MT-PG1VT, PG1YT-PG3YT, PG5RT-PG6NT, PGA-PGW, PH1AT, PH1FT, PH1UT, PH2ST, PH3YT, PH4AT, PH5GT, PH6GT, PH7FT, PH7UT, PH9AT, PH9ST, PH900, PHA-PHW, PJ2AT, PJ2CT, PJ2HT, PJ3-PJ4, PJ5AN, PJA-PJW, PW, PXA-PXX, QAA25, QAB00-QAB05, QAB99, QAC, QAD20, QAE-QAF, QAG10-QAG99, QAJ, QBA25, QBB00-QBB05, QBB99, QBC,

QBD20, QBE, QBG10-QBG99, QBJ, QCA25, QCA30, QCB00-QCB05, QCB99, QCC, QCD20, QCE-QCG, QCJ, QDA25, QDB00-QDB05, QDB99, QDC, QDD20, QDE, QDG10-QDG99, QDJ, QXA25, QXB00-QXB05, QXB99, QXC, QXD20, QXE, QXG10-QXG99, QXJ, QX2ZT, QX3AT, QX3CT, QX3LT, QX3YT, QX4, S, TAA-TAD, TAW99, TA100, TBA-TJF, TJG10, TJJ, TJL-TJW, TK-TL, TMA-TPX, TQA-TQD, TQW00, TQW02, TQW30-40, TQW99, TQX00-TQX10, U, WW20, WW30-WW31, WW40, WW50, WX100-WX105, WX140-WX144, WX7-WX9, XCC00, XFE00, XFN96, XFX00, XFX10, XFX20, XFX97, XJW99, XPX00, XPX04, XPX08, XPX99, XW000, XW1-XW5, XX1AT, XX1BT, XX1CT, XX1DT, XX1XT, XX2AT-XX2DT, XX2XT, XX3AT-XX3DT, XX3XT, XX4-XX7, Y, ZC-ZP, ZS-ZX, ZZ

For peer review only

STROBE 2007 (v4) Statement—Checklist of items that should be included in reports of *cohort studies*

Section/Topic	Item #	Recommendation	Reported on page #
**Title and abstract**	1	(*a*) Indicate the study's design with a commonly used term in the title or the abstract	#1
		(*b*) Provide in the abstract an informative and balanced summary of what was done and what was found	#2-3
**Introduction**			
Background/rationale	2	Explain the scientific background and rationale for the investigation being reported	#4-5
Objectives	3	State specific objectives, including any prespecified hypotheses	#4-5
**Methods**			
Study design	4	Present key elements of study design early in the paper	#5-6
Setting	5	Describe the setting, locations, and relevant dates, including periods of recruitment, exposure, follow-up, and data collection	#5-6
Participants	6	(*a*) Give the eligibility criteria, and the sources and methods of selection of participants. Describe methods of follow-up	#6
		(*b*) For matched studies, give matching criteria and number of exposed and unexposed	NA
Variables	7	Clearly define all outcomes, exposures, predictors, potential confounders, and effect modifiers. Give diagnostic criteria, if applicable	#5-6
Data sources/ measurement	8*	For each variable of interest, give sources of data and details of methods of assessment (measurement). Describe comparability of assessment methods if there is more than one group	*#5-6*
Bias	9	Describe any efforts to address potential sources of bias	#5-6 and discussion section #13
Study size	10	Explain how the study size was arrived at	#5-6
Quantitative variables	11	Explain how quantitative variables were handled in the analyses. If applicable, describe which groupings were chosen and why	#5-7
Statistical methods	12	(*a*) Describe all statistical methods, including those used to control for confounding	#6-7
		(*b*) Describe any methods used to examine subgroups and interactions	#6-8
		(*c*) Explain how missing data were addressed	NA
		(*d*) If applicable, explain how loss to follow-up was addressed	NA

For peer review only

		(*e*) Describe any sensitivity analyses	#7
**Results**			
Participants	13*	(a) Report numbers of individuals at each stage of study—eg numbers potentially eligible, examined for eligibility, confirmed eligible, included in the study, completing follow-up, and analysed	#8-9
		(b) Give reasons for non-participation at each stage	NA
		(c) Consider use of a flow diagram	NA
Descriptive data	14*	(a) Give characteristics of study participants (eg demographic, clinical, social) and information on exposures and potential confounders	#9
		(b) Indicate number of participants with missing data for each variable of interest	NA
		(c) Summarise follow-up time (eg, average and total amount)	NA
Outcome data	15*	Report numbers of outcome events or summary measures over time	#8-9
Main results	16	(*a*) Give unadjusted estimates and, if applicable, confounder-adjusted estimates and their precision (eg, 95% confidence interval). Make clear which confounders were adjusted for and why they were included	#figure 1
		(*b*) Report category boundaries when continuous variables were categorized	NA
		(*c*) If relevant, consider translating estimates of relative risk into absolute risk for a meaningful time period	NA
Other analyses	17	Report other analyses done—eg analyses of subgroups and interactions, and sensitivity analyses	#9-11
**Discussion**			
Key results	18	Summarise key results with reference to study objectives	#11-12
**Limitations**			
Interpretation	20	Give a cautious overall interpretation of results considering objectives, limitations, multiplicity of analyses, results from similar studies, and other relevant evidence	#11-14
Generalisability	21	Discuss the generalisability (external validity) of the study results	#11-12
**Other information**			
Funding	22	Give the source of funding and the role of the funders for the present study and, if applicable, for the original study on which the present article is based	#15

*Give information separately for cases and controls in case-control studies and, if applicable, for exposed and unexposed groups in cohort and cross-sectional studies.

For peer review only
